# A High-Speed Visual BCI Based on Hybrid Frequency–Phase–Space Encoding and High-Density EEG Decoding

**DOI:** 10.34133/cbsystems.0555

**Published:** 2026-03-26

**Authors:** Gege Ming, Weihua Pei, Sen Tian, Xiaogang Chen, Xiaorong Gao, Yijun Wang

**Affiliations:** ^1^ Department of Biomedical Engineering, Tsinghua University, Beijing 100084, China.; ^2^ Laboratory of Solid-State Optoelectronics Information Technology, Institute of Semiconductors, Chinese Academy of Sciences, Beijing 100083, China.; ^3^ School of Future Technology, University of Chinese Academy of Sciences, Beijing 100049, China.; ^4^ Suzhou Nianji Intelligent Technology Co., Ltd., Suzhou 215133, China.; ^5^ Institute of Biomedical Engineering, Chinese Academy of Medical Sciences and Peking Union Medical College, Tianjin 300192, China.; ^6^ Chinese Institute for Brain Research, Beijing 102206, China.

## Abstract

Brain–computer interface (BCI) technology establishes a direct communication pathway between the brain and external devices. Current visual BCI systems suffer from insufficient information transfer rates (ITRs) for practical use. Spatial information, a critical component of visual perception, remains underexploited in existing systems because the limited spatial resolution of recording methods hinders the capture of the rich spatiotemporal dynamics of brain signals. This study proposed a hybrid frequency–phase–space encoding method, integrated with high-density electroencephalogram (EEG) recordings, to develop high-speed BCI systems. EEG data were recorded using a 256-channel standard cap, and 4 electrode configurations comprising 66, 32, 21, and 9 parieto-occipital electrodes, extracted from 256-, 128-, and 64-channel caps (abbreviated as 66/256, 32/128, 21/64, and 9/64), were systematically compared. In the classical frequency–phase encoding the 40-target BCI paradigm, the 66/256, 32/128, and 21/64 electrode configurations brought theoretical ITR increases of 83.66%, 79.99%, and 55.50% over the traditional 9/64 setup. In the proposed frequency–phase–space encoding 200-target BCI paradigm, these increases climbed to 195.56%, 153.08%, and 103.07%, respectively. The online BCI system achieved an average actual ITR of (472.72 ± 15.06) bits per minute. Taken together, these findings clarify how the spatiotemporal encoding strategy and electrode density jointly determine achievable ITRs and provide quantitative design guidelines for future high-speed visual BCIs.

## Introduction

The visual evoked potential (VEP)-based brain–computer interface (BCI) system is a noninvasive neural interaction technology that interprets brain activity elicited by visual stimuli, enabling the control of external devices or the transmission of information [1–25]. With its high information transfer rate (ITR), rapid responsiveness, and minimal training requirements for users, the VEP-BCI system has attracted considerable attention in both academic research and consumer applications [26,27]. With the rapid advancement of VEP encoding and decoding methods, a high-speed BCI speller reported in 2018 demonstrated the ability to input 1 character every 0.8 s (stimulus duration: 0.3 s, interstimulus interval: 0.5 s, accuracy: 89.83%), achieving an actual ITR of 325.33 bits per minute (bpm), which at the time was the highest reported value for noninvasive BCIs [2]. Despite these achievements, the ITR of current BCI technologies remains far from matching the efficiency of traditional human–computer interaction methods, such as touchscreens and keyboards. Moreover, since 2018, progress in improving the ITR of VEP-based BCIs has nearly stagnated, underscoring the urgent need for innovations in the field [1–23].

Existing research on visual BCIs predominantly employs commercially available 64-channel electrode caps adhering to the international 10-10 system, which provide a spatial resolution of approximately 3 cm [1–14,16–18,28–33]. However, the fundamental structural and functional unit of the visual cortex occupies only approximately 1 mm^2^ of cortical surface area and comprises a complete orientation column cycle, a full set of left- and right-eye ocular dominance columns, and blobs [34]. Previous theoretical and experimental studies on the spatial Nyquist limit of scalp electroencephalogram (EEG)—defined as the maximum spatial detail that can be resolved without aliasing for a given interelectrode spacing—indicate that approximately 128 electrodes are sufficient to adequately sample signals across the entire scalp [35,36]. However, the objectives of EEG analysis often extend beyond capturing high-amplitude, low-spatial-frequency signals. Many applications require detecting finer neural activity within the visual cortex. Petrov et al. [37] demonstrated that employing a custom ultrahigh-density grid electrode array to reduce the interelectrode spacing in the parieto-occipital scalp region (from 3 to 1 cm) effectively improved the signal-to-noise ratio (SNR) of VEP responses. Similarly, Robinson et al. [38] modified a 128-channel electrode cap based on the Biosemi system by increasing the electrode density in the occipital and temporal regions by 2 to 3 times. Their findings confirmed a clear advantage of ultrahigh-density electrodes (1.4 cm interelectrode spacing) over conventional commercial electrodes (2.8 cm interelectrode spacing) in capturing subtle spatial patterns of visually evoked EEG activity. These spatial characteristics are crucial for improving the decoding of visual information and hold promise for enhancing the communication speed of BCI systems.

The steady-state visual evoked potential (SSVEP) refers to the stable response of the visual system elicited by periodic repetitive stimuli [31]. Among EEG-based BCI systems, SSVEP-BCI systems exhibit the highest performance [2,8]. Encoding methods for SSVEP-BCI systems are generally categorized into 2 main types: single-feature encoding and hybrid-feature encoding [24]. Single-feature encoding includes methods such as frequency modulation [17], phase modulation [23], space modulation [10], and so on. These approaches utilize the time-locked and phase-locked characteristics of SSVEP signals, along with the retinotopic mapping properties of the visual system, to encode different commands in BCI systems based on the temporal and spatial features of visual stimuli. Compared to other encoding methods, current spatial encoding strategies exhibit notable limitations in the size of visual stimuli, the number of encodable targets, and overall system performance [10,13,15]. For instance, Chen et al. [17] designed a 45-target (3.5° visual angle) SSVEP-BCI speller system that utilized a frequency range of 7 to 15.8 Hz with 0.2-Hz intervals, achieving an actual ITR of 105 bpm (stimulus duration: 2 s, interstimulus interval: 0.3 s, accuracy: 84.1%). In contrast, Maye et al. [15] developed a 9-target BCI system employing the relative positional differences between circular flickering stimuli (27° visual angle) and the fixation point, resulting in an actual ITR of only 40.80 bpm (stimulus duration: 3 s, interstimulus interval: 1 s, accuracy: 95%). Hybrid-feature encoding integrates multiple features to achieve more efficient encoding of visual targets [32]. For example, Nakanishi et al. [2] combined phase information with the frequency encoding method to enhance the differentiation of SSVEP responses between adjacent frequencies. This approach enabled the development of a 40-target SSVEP-BCI system that achieved an actual ITR of 325.33 bpm, which at the time was the highest reported value for noninvasive BCIs [2]. While the reported spelling speed of 0.8 s (stimulus duration: 0.3 s, interstimulus interval: 0.5 s) per character in this study approached the upper limit of human gaze control, substantial room for further improvement remained, particularly in terms of increasing the number of commands (40) and improving classification accuracy (89.80%).

The visual system possesses remarkably high spatial resolution, with spatial location information consistently preserved throughout the transmission of visual information [39]. A distinct retinotopic projection exists between the primary visual cortex of each cerebral hemisphere and the corresponding hemiretina of the contralateral visual field [40,41]. As a result, variations in the relative position between visual stimuli and the focus of overt attention produce unique topographical distributions of scalp EEG signals [4,10,13]. However, traditional 64-channel EEG recordings exhibit notable limitations in decoding spatial information, hindering the advancement of efficient encoding strategies that seamlessly integrate spatial information with other features such as frequency and phase.

This study aimed to enhance the ITR of visual BCI systems by jointly exploiting the spatiotemporal properties of visual stimulation and high-density EEG recordings. On the one hand, the command set was expanded by assigning multiple fixation points as independent targets to visual stimuli flickering at specific frequencies and initial phases. On the other hand, a 256-channel EEG acquisition system was employed to capture high-spatial-resolution EEG signals and provide richer spatial information for decoding. The key novelties of this work were twofold. First, a hybrid frequency–phase–space encoding paradigm was proposed and validated, which explicitly exploits the spatiotemporal structure of SSVEP responses to fully leverage the advantages of high-density EEG. Second, the influence of electrode density on the performance of frequency–phase information decoding and spatial information decoding was systematically and quantitatively characterized across 4 electrode configurations (9/64, 21/64, 32/128, and 66/256) derived from standard EEG caps. Together, these contributions provide data-driven guidance for the design of large-command-set, high-speed visual BCIs.

## Materials and Methods

### Subjects and signal acquisition

#### Participants and experimental environment

A total of 15 healthy participants (including 8 females, aged 23 to 34 years) with normal or corrected-to-normal vision were enrolled in the study. Written informed consent was obtained from all participants prior to the experiment, and they were fully briefed on the procedures and requirements. The protocol conformed to the Declaration of Helsinki and was approved by the Institutional Review Board of Tsinghua University (No. 20230058). All experiments took place in a brightly lit room with stable lighting conditions. Participants were seated comfortably on a chair positioned approximately 70 cm directly in front of the screen. To reduce electromyographic artifacts caused by head movements, a chin rest was used to support their chin and forehead.

#### High-density EEG acquisition

**Fig. 1.**
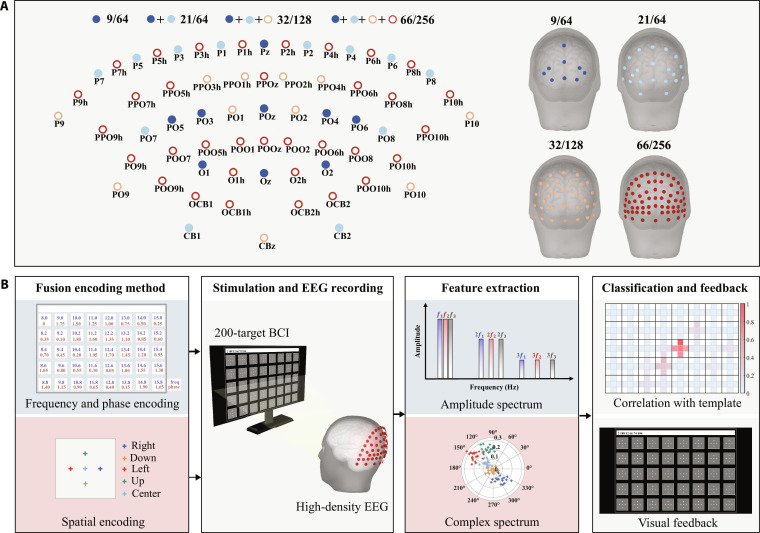
Schematic overview of the 200-target brain–computer interface (BCI) system. (A) Top and rear views of 9/64, 21/64, 32/128, and 66/256 electrode configurations. (B) Basic structure of the BCI system. The 200 targets were formed by combining 40 square luminance-modulated patches (“flickers”) with distinct frequency–phase values and 5 fixation points (right, down, left, up, and center) embedded within each flicker. Each square patch flickered periodically according to its assigned waveform, whereas the fixation markers remained at constant luminance. Stimuli with high visual resolution were presented to the subjects, while high spatial resolution electroencephalogram (EEG) was recorded for feature extraction. The classification results were presented to the subjects in the form of visual feedback, including color changes of selected fixation points and the display of corresponding numeric labels (Fig. S1 and Movie S1) in the text input box.

Because periodic visual flicker elicits strong SSVEPs in the parieto-occipital cortex [3,16,17], a commercial 256-channel Quik-Cap Neo Net cap (Compumedics Neuroscan) configured according to the international 10-5 system was employed [29], and 66 channels over the parieto-occipital region (Fig. 1A, created using brainstorm [42]), were selected for decoding. The cap was equipped with passive, gel-based wet electrodes with a mean interelectrode distance of 1.5 cm. The reference electrode was positioned at the vertex and the ground electrode was located at the midpoint of the FPz–Fz line. During data acquisition, all electrode impedances were maintained below 15 kΩ, and the sampling rate was set to 1,000 Hz. At the onset of each visual stimulation, event triggers were generated by the stimulation program, sent to the EEG amplifier via a parallel port, and then recorded in an event channel synchronized with the EEG data. In subsequent data analysis, channel selection was used to generate standard Quik-Cap configurations with lower electrode density, such as 128 channels (mean interelectrode distance: 2.0 cm, parieto-occipital channel number: 32) and 64 channels (mean interelectrode distance: 2.8 cm, parieto-occipital channel number: 21). This study systematically compared the visual information decoding the performance of 4 electrode configurations: 66/256, 32/128, 21/64, and 9/64, where the numerator denotes the number of parieto-occipital electrodes used for decoding and the denominator denotes the total number of channels in the corresponding EEG cap. The 9/64 configuration corresponded to 9 electrode positions (Pz, POz, Oz, PO3 to PO6, O1, and O2) from the standard 64-channel Quik-Cap, which are commonly used as a baseline montage in previous visual BCI studies [2,3,6,8,11,16,30–32].

### BCI paradigm

#### Hybrid frequency–phase–space encoding method

To explore the potential of high-density EEG in decoding spatiotemporal information, a visual BCI system was designed in which targets were encoded by both rhythmic luminance modulation and the spatial position of the fixation point relative to each flicker. The structure diagram of the 200-target BCI system is depicted in Fig. 1B. This study proposed a hybrid frequency–phase–space encoding strategy to enhance the visual resolution requirements in decoding tasks, thereby fully leveraging the advantages of high spatial resolution EEG recordings. The hybrid frequency–phase–space encoding method generated 200 targets through a combination of 40 flickers and 5 fixation points. First, following the frequency–phase encoding method [32], the 40 flickers were arranged in a 5 × 8 matrix, with each flicker measuring 144 × 144 pixels, corresponding to a visual angle of 3.3°. The horizontal and vertical spacing between adjacent flickers was 50 pixels. As illustrated in Fig. 1B, each flicker was assigned progressively increasing frequency and phase values, arranged sequentially from top to bottom and left to right. The frequency parameters ranged from 8 to 15.8 Hz with 0.2-Hz intervals, while the phase parameters varied from 0 to 2π with 0.35π intervals. Unlike traditional high-speed BCI systems that assign a unique flicker to each target [2,5,17,32], this study further introduced a spatial coding strategy. A total of 5 cross-shaped fixation points (color: white) were positioned along the horizontal centerline (left and right fixation points), the vertical centerline (up and down fixation points), and the center (center fixation point) of each flicker. The distances between the right, down, left, and up fixation points and the center fixation point were uniformly 36 pixels, equivalent to 25% of the flicker’s edge length. Each fixation point occupied a visual angle of 0.23°. This hybrid encoding strategy allowed the system to multiplicatively increase the number of targets to 200, while maintaining the same interface size. The system interface was presented on a 24.5-inch liquid crystal display (Alienware 2521H) with a resolution of 1,920 × 1,080 pixels and a refresh rate of 240 Hz. The brightness values of the flickers were modulated for each frame using the sampled sinusoidal stimulation method [17]. The stimulation program was developed with Psychophysics Toolbox under MATLAB [43].

#### Target classification algorithm

As summarized in Fig. 1B, target decoding in the 200-target BCI system relied on the spatiotemporal structure of SSVEP responses. SSVEPs exhibit strong time-locking characteristics with the periodic flicker stimuli. Its spectral features are marked by prominent peaks at the stimulus frequency and its harmonics, which serve as key features to distinguish different flickers [3]. In addition, the one-to-one spatial mapping exists between retinal positions and the corresponding visual cortical locations [4], enabling the SSVEP responses induced by different fixation points exhibiting distinct amplitude and phase topographic distributions. The use of high-density EEG further enhances the ability to capture these subtle spatial pattern differences. These properties were exploited through task-discriminative spatiotemporal filtering and template matching, corresponding to the “Feature extraction” and “Classification and feedback” stages in Fig. 1B.

In this study, task-discriminant component analysis (TDCA), a state-of-the-art algorithm for SSVEP decoding, was used to derive a shared set of optimal spatiotemporal filters across all targets. For training data, the EEG of the *i*th trial for a given target is denoted as $X^{\left(i\right)}\in {\mathbb{R}}^{N_{c}\times N_{p}}$, where $i=1,2,\cdots N_{b}$, $N_{c}$ is the number of electrodes, $N_{p}$ is the number of data points per trial (sampling rate multiplied by the decoding-window length), and $N_{b}$ is the number of training trials per target. First, for each trial, the multichannel EEG data are augmented as follows:

$$\tilde{X}={\left[X^{T},\;X_{1}^{T},\cdots ,\;X_{l}^{T}\right]}^{T}$$ (1)

where $X_{l}$ represents the EEG data within the time window [1 +l, $N_{p}+l$], and 𝑙 represents the delay order. The augmented EEG data segment is denoted as $\tilde{X}\in {\mathbb{R}}^{\left(l+1\right)N_{c}\times N_{p}}$. The same processing procedure was applied to the testing data, with the only difference being that data points exceeding $N_{p}$ were zero-padded. Furthermore, by incorporating prior knowledge of the stimulus frequency in SSVEPs, both the training and testing EEG data undergo secondary augmentation:

$$X_{a}=\left[\tilde{X},{\tilde{X}}_{p}\right]$$ (2)

where the subscript “a” indicates augmented data, and ${\tilde{X}}_{p}$ represents the projection of $\tilde{X}$ onto the subspace spanned by the sine-cosine reference signals. For the nth target, the template signal was obtained by averaging the training trials:

$${\bar{X}}_{n}=\frac{1}{N_{b}}\sum_{i=1}^{N_{b}}X_{a}^{\left(i\right)}$$ (3)

where $n=1,2,\cdots N_{t}$, and $N_{t}$ represents the number of targets in the classification task. Subsequently, the common projection direction W for all targets was determined based on the Fisher criterion:

$$\underset{W}{\text{max}}\frac{\mathrm{tr}\left[W^{T}H_{b}H_{b}^{T}W\right]}{\mathrm{tr}\left[W^{T}H_{w}H_{w}^{T}W\right]}$$ (4)

where $H_{b}$ and $H_{w}$ denote the between-class and within-class difference matrix constructed from the secondary augmented data $X_{a}$. A more detailed description of the process can be found in the paper by Liu et al. [8]. To fully leverage the fundamental and harmonic components of the SSVEP response, the raw EEG signal was decomposed into multiple subband components using a filter bank [3]. The spatiotemporal filter $W^{\left(m\right)}$ for the mth subband (where m=1, 2, …, $N_{m}$, and $N_{m}$ represents the number of subbands) was then computed. Before calculating the correlation coefficient between the individualized template for the nth target and a single testing trial, the template signal ${\bar{X}}_{n}^{\left(m\right)}$ and testing data $X^{\left(m\right)}$ were dimensionally reduced using spatiotemporal filters:

$$r_{n}^{\left(m\right)}=\rho \left({\left(X^{\left(m\right)}\right)}^{T}W^{\left(m\right)},{\left({\bar{X}}_{n}^{\left(m\right)}\right)}^{T}W^{\left(m\right)}\right)$$ (5)

where $\rho \left(a,b\right)$ denotes the Pearson correlation coefficient between ***a*** and ***b***. The weighted sum of the squared correlation coefficients across all subbands is defined as the classification feature:

$$\rho_{n}=\sum_{m=1}^{N_{m}}f\left(m\right)\cdot {\left(r_{n}^{\left(m\right)}\right)}^{2}$$ (6)

where the weight coefficient is given by $f\left(m\right)=m^{-1.25}+0.25\;$ [3]. Finally, the classification result was obtained by selecting the class with the highest $\rho_{n}$:

$$\tau =\underset{n}{\text{arg}\;\text{max}}\rho_{n}\;$$ (7)

Additionally, the classical task-related component analysis (TRCA) [2] and EEGNet [44,45] algorithms were also employed for target recognition in this study to demonstrate that the benefits of high-density EEG reflect a general increase in information content that can be exploited by different decoders. The parameter settings for both algorithms strictly followed the corresponding original publications.

### Experimental procedure

This study involved offline and online experiments conducted on separate days. A total of 15 subjects participated in the offline experiment, and 10 of them remained available and consented to join the follow-up online experiment. During the offline experiment, all participants utilized the 200-target BCI system. Each participant completed 18 blocks of EEG data collection, with each block randomly traversing all 200 targets, corresponding to 200 trials. That is, 18 trials of EEG data were obtained for each target. Each trial consisted of 0.5 s of cue time (a fixation point among the 200 targets turned red and the corresponding flicker was highlighted with a 15-pixel-wide red frame), followed by 0.5 s of stimulation (40 flickers flashed periodically and simultaneously). The 0.5-s cue duration was set in accordance with previous high-speed SSVEP-BCI studies to provide sufficient time for target search and gaze shifting [1,2]. The offline data were used to determine the optimal target number and fixation combination for each participant.

The online experiment consisted of a stimulation duration optimization session, a training session, and a testing session. During the stimulation duration optimization session, all participants uniformly used an 80-target BCI system, comprising 40 flickers and 2 fixation points (down and up), to streamline the optimization process. Given that the peak actual ITR in the offline experiment occurred at a data length of 0.2 s, and previous studies have shown that participants can adapt to a stimulation time of 0.3 s [2], the available stimulation duration for adjustment were 0.2, 0.25, and 0.3 s. First, 6 blocks of EEG data were collected under a 0.5-s cue time and a 0.2-s stimulation time. The classification performance for the 80 targets was then compared with the results obtained under the same conditions in the offline experiment. If the performance difference exceeded 10%, the stimulation duration would be extended to 0.25 s and another 6 blocks of EEG data would be collected. If a substantial performance difference still remained, the stimulation duration of 0.3 s would be directly adopted in subsequent experiments. During the training session, the system parameters were configured based on each participant’s personalized target number, fixation combination, and stimulation duration. A total of 18 blocks of EEG data were collected, with each block randomly traversing all targets. The cue time was maintained at 0.5 s. During the testing session, the experimental procedure was similar to that of the training session, with the primary difference being the provision of visual feedback to participants when the online data analysis program obtained the classification results. The visual feedback consisted of color changes of the selected fixation points and the display of corresponding numeric labels in the text input box (Fig. 1B and Fig. S1). Each participant completed 5 blocks of EEG data collection in this session.

### Data analysis

#### BCI performance evaluation

EEG data segments from 66 channels were extracted based on a 140-ms visual latency and subsequently down-sampled to 250 Hz. Target classification was performed using the filter bank TDCA algorithm (delay order l set to 4, with 5 subbands and passband frequency ranges set to [6 Hz, 90 Hz], [14 Hz, 90 Hz], [22 Hz, 90 Hz], [30 Hz, 90 Hz], and [38 Hz, 90 Hz]). These subbands were designed by setting the lower cutoff of the mth band to $m\times f_{\min}-$2 Hz (with $f_{\min}=$8 Hz as the minimum stimulus frequency) and fixing the upper cutoff at 90 Hz, following the filter-bank scheme in Ref. [3]. For the offline BCI experiment, a leave-one-out cross-validation approach was employed, where each of the 18 blocks was held out in turn as the test set while the remaining blocks served as the training set. This method was used to calculate the classification accuracy for varying target numbers (ranging from 40 to 200) and different fixation combinations (Table 1). The data lengths used for classification ranged from 0.02 to 0.1 s with a step size of 0.02 s, and from 0.1 to 0.5 s with a step size of 0.1 s. For the online BCI experiment, the TDCA spatiotemporal filter and individual templates calculated using EEG data from the training session were applied to the testing session. In addition to classification accuracy, ITR was calculated using Eq. (8), where N represents the number of targets (ranging from 40 to 200), P represents the classification accuracy, and T represents the time required for each target selection. For the actual ITR calculation, T includes both the stimulation time and the cue time; for the theoretical ITR calculation, T includes only the stimulation time.

$$\mathrm{ITR}=\left(\log_{2}N+P\log_{2}P+\left(1-P\right)\log_{2}\left(\frac{1-P}{N-1}\right)\right)\times \frac{60}{T}$$ (8)

**Table 1.** All combinations of fixation points under different target numbers

Combination label	1 fixation point	2 fixation points	3 fixation points	4 fixation points	5 fixation points
1	Right	Right, down	Left, up, center	Right, down, left, up	Right, down, left, up, center
2	Down	Right, left	Down, up, center	Right, down, left, center	–
3	Left	Right, up	Down, left, center	Right, down, up, center	
4	Up	Right, center	Down, left, up	Right, left, up, center	
5	Center	Down, left	Right, up, center	Down, left, up, center	
6	–	Down, up	Right, left, center	–	
7		Down, center	Right, left, up		
8		Left, up	Right, down, center		
9		Left, center	Right, down, up		
10		Up, center	Right, down, left		
Target number	40	80	120	160	200

#### Signal feature analysis

After averaging the 18 blocks of EEG data segments recorded during offline experiments, a fast Fourier transform (FFT) was applied to the 66 electrode positions to obtain the amplitude and phase spectra. The SNR is then calculated as the ratio of signal strength to noise strength:

$$\begin{array}{c} \mathrm{SNR}=20\;\log_{10}\frac{10\cdot y\left(f\right)}{\sum_{k=1}^{5}\left[y\left(f-\Delta f\cdot k\right)+y\left(f+\Delta f\cdot k\right)\right]} \end{array}$$ (9)

where signal strength is defined as the amplitude value $y\left(f\right)$ at the target frequency f, while noise strength is defined as average amplitude value of the 10 frequencies (5 frequencies on each side) adjacent to the target frequency f. Although the data length was only 0.5 s, zero-padding was applied prior to the FFT, resulting in a frequency resolution Δf of 1 Hz. SNR and phase topographies were subsequently plotted to assess differences in response intensity and phase across various electrode positions, as well as to explore the relationship between fixation point locations and the spatial distribution characteristics of EEG responses. In addition, the 66-channel EEG data segments can be projected into one-dimensional space using the TDCA spatiotemporal filter, effectively suppressing background EEG noise while achieving data dimensionality reduction. Therefore, after applying the TDCA spatiotemporal filter to the 18 blocks of EEG data segments, FFT was performed again, and the differences in amplitude and phase distributions across different fixation points were visualized using a more intuitive method (complex spectra). Additionally, for the raw data filtered with the [6 Hz, 90 Hz] bandpass filter, the Pearson correlation coefficient between each pair of the 66 channels was calculated to assess the contribution of high-density EEG acquisition.

#### Greedy search for optimal electrode combination

A greedy search strategy was employed to obtain the optimal electrode combination and its corresponding classification performance for a given electrode number under a specific electrode configuration. Because most subjects achieved their maximum actual ITR with 80 targets, the optimization was performed on the 80-target offline classification task with a data length of 0.2 s. The initial electrode sets corresponded to the 3 configurations used in this study: 21 electrodes from the 64-channel cap (21/64), 32 electrodes from the 128-channel cap (32/128), and 66 electrodes from the 256-channel cap (66/256). Firstly, we initialized the current electrode set as C, containing all electrodes in a given configuration, and denoted its size by $N_{c}$. Next, at each iteration, we generated all $N_{c}$ candidate subsets by removing exactly one electrode from C, so that each candidate subset contained $N_{c}-1$ electrodes. For every candidate subset, we computed the mean classification accuracy across the 15 subjects in the 80-target task. The subset with the highest mean accuracy was then assigned to C, and $N_{c}$ was updated accordingly. This iterative procedure was repeated until only 2 electrodes remained. Finally, for each electrode count, the best-performing subset and its associated accuracy were recorded. This procedure yielded the optimal electrode combination and its corresponding classification performance as a function of the number of electrodes.

#### Dynamic window classification based on TDCA

Reducing the stimulus duration for trials with high signal quality minimizes the time spent on target selection, while extending the stimulus duration for trials with poor signal quality improves classification accuracy. Accordingly, a dynamic window classification strategy was employed to determine the optimal output time window for achieving higher ITRs. As the time window length increases, the probability of correctly classifying the target in a single trial also rises. Based on this observation, a confidence factor $c\left(k\right)={\left(k/50\right)}^{2}$ was introduced to adjust the output probability across different time window lengths, where k=1, 2, …, $N_{k}$, and $N_{k}$ represents the number of time windows. The confidence factor was applied to the weighted correlation coefficients between the individualized template for the nth target and a single testing trial:

$$\rho_{\mathrm{tn}}=c\left(k\right)*\sum_{m=1}^{N_{m}}f\left(m\right)\cdot {\left(r_{n}^{\left(m\right)}\right)}^{2}$$ (10)

where $r_{n}^{\left(m\right)}$ is the correlation coefficient between the individualized template for the 𝑛th target and the test trial in the mth subband [see Eq. (5)], $N_{m}$ is the number of subbands, and $f\left(m\right)$ = $m^{-1.25}+0.25$ [3] is used to compute the weight coefficient of the mth subband. The classification result was generated only when the risk cost of the output fell below a predefined threshold, defined as:

$$T\left(s\right)>-\;\left(\rho_{\max}-\rho_{2\text{nmax}}\right)\;$$ (11)

where $\rho_{\max}$ and $\rho_{2\text{nmax}}$ represent the largest and second-largest correlation coefficients among all targets, respectively. In this study, the dynamic window classification algorithm was validated on offline experimental data. The threshold $T\left(s\right)$ was defined as $T\left(s\right)=-\left(s\times 10^{-5}\right)/2$, where s=1, 2, ⋯, $N_{s}$, and $N_{s}$ represents the number of threshold values. The time window range was set between 0.1 and 0.5 s with an interval of 0.1 s, resulting in $N_{k}$ equal to 5. The threshold range was set from −0.5 × 10^−5^ to −2.5 × 10^−4^, with $N_{s}$ equal to 50. Specifically, if the data length extended to 0.5 s without satisfying the threshold, the classification result based on the 0.5-s data length would be adopted.

#### Statistical analysis

This study used repeated-measures analysis of variance (RMANOVA) to assess whether the differences across various experimental conditions were statistically significant. Prior to analysis, the normality and sphericity assumptions of the data were checked. If the sphericity assumption was violated, the Greenhouse–Geisser correction was applied. Specifically, one-way RMANOVA was used to compare the classification performance across different fixation combinations. When statistically significant differences were observed, post-hoc pairwise comparisons were conducted with Bonferroni correction to control for Type I errors. Two-way RMANOVA was used to examine the effects of electrode density and data length on classification performance, including main effects and interaction effects. A significant interaction effect indicated that the effect of one factor varied depending on the level of the other factor; in such cases, a simple main effects analysis was conducted using one-way RMANOVA to further explore the differences at various levels. The P values reported in this study were adjusted using Bonferroni correction.

## Results

### High-density EEG substantially enhances the performance of BCI

#### Offline BCI performance

**Fig. 2.**
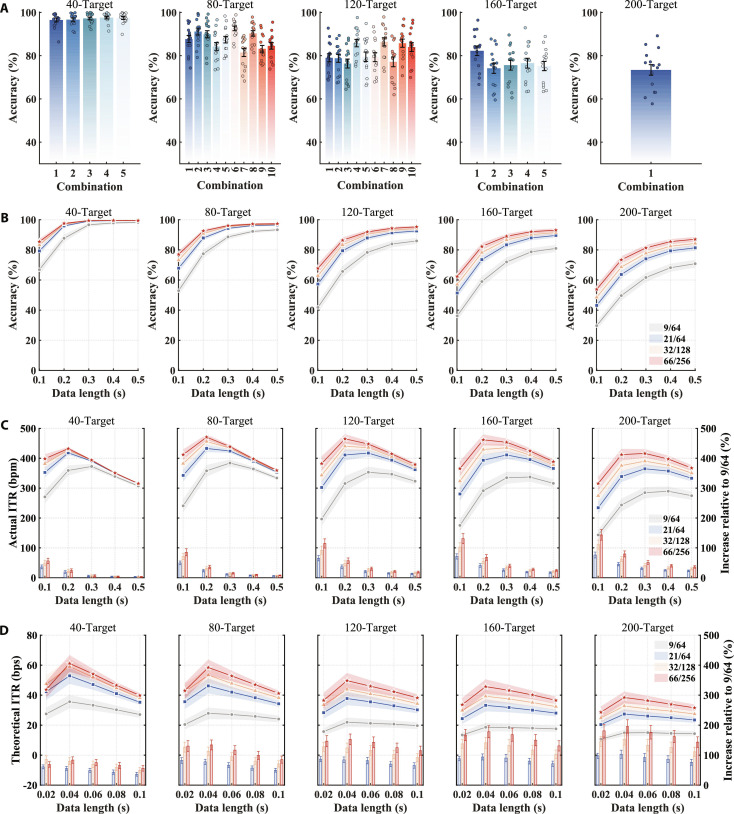
Increasing electrode density substantially enhances visual information decoding performance. (A) Classification accuracy for all possible fixation combinations using electrode configuration of 66/256 and data length of 0.2 s. Error bars indicate standard errors, while discrete circles denote the individual results of 15 subjects. (B) Classification accuracy of optimal fixation combinations for 4 electrode configurations with data lengths ranging from 0.1 to 0.5 s. (C) Actual ITR (lines) for 4 electrode configurations, along with the actual ITR increase relative to the 9/64 electrode configuration (bars) for the 21/64, 32/128, and 66/256 configurations with data lengths ranging from 0.1 to 0.5 s. (D) Theoretical ITR (lines) for 4 electrode configurations, along with the theoretical ITR increase relative to the 9/64 configuration (bars) with data lengths ranging from 0.02 to 0.1 s. The relative increase is defined as the ratio of the difference in ITR between a given configuration and the 9/64 configuration to the ITR of the 9/64 configuration. The shaded area and error bars indicate the standard errors (number of participants = 15).

The number of fixation points placed on each flicker can be varied from 1 to 5, enabling the implementation of BCI systems with 40 to 200 targets. Table 1 presents all possible fixation combinations for different target numbers. An offline BCI experiment was conducted to determine the optimal number and placement of fixation points. A total of 15 participants took part in the experiment. The filter-bank task TDCA algorithm, combined with leave-one-out cross-validation, was applied for target recognition. Under the electrode configuration of 66/256 and data length of 0.2 s, the classification tasks for 40, 80, 120, 160, and 200 targets achieved the highest accuracy of 97.58% (combination 4: up), 92.59% (combination 6: down and up), 86.38% (combination 7: right, left, and up), 82.26% (combination 1: right, down, left, and up), and 73.40% (combination 1: right, down, left, up, and center), respectively (Fig. 2A and Fig. S2). For the 40-target BCI system without space encoding, the one-way RMANOVA showed no significant performance differences across the 5 fixation points [*F*(2.33, 32.66) = 3.01, P = 0.051, partial *η*^2^ = 0.18]. For the 80-target [*F*(3.88, 54.29) = 38.69, P = 4.23 × 10^−15^, partial *η*^2^ = 0.73], 120-target [*F*(3.27, 45.80) = 56.84, P = 5.47 × 10^−16^, partial *η*^2^ = 0.80], and 160-target [*F*(4, 56) = 70.01, P = 3.95 × 10^−21^, partial *η*^2^ = 0.83] BCI systems, which considered 2 to 4 fixation positions, significant performance differences were observed across different fixation combinations (Fig. S3A).

Based on the optimal fixation combinations, the classification accuracy under different electrode densities was further analyzed (Fig. 2B). As assessed by 2-way (density × data length) RMANOVA, the interaction between electrode density and data length was statistically significant for all target numbers [40-target: *F*(2.10, 29.35) = 70.02, P = 7.14 × 10^−59^, partial *η*^2^ = 0.83; 80-target: *F*(2.39, 35.87) = 106.01, P= 1.02 × 10^−16^, partial *η*^2^ = 0.88; 120-target: *F*(2.39, 33.52) = 78.35, P= 3.43 × 10^−14^, partial *η*^2^ = 0.85; 160-target: *F*(2.47, 34.64) = 51.18, P = 5.59 × 10^−12^, partial *η*^2^ = 0.79; 200-target: *F*(2.06, 28.84) = 20.42, p = 3 × 10^−6^, partial *η*^2^ = 0.59]. In the frequency–phase encoding BCI system with 40 targets, paired *t* tests with Bonferroni correction (number of comparisons = 6) showed that the 66/256 electrode configuration significantly outperformed the other 3 configurations when the data length was less than or equal to 0.2 s (P < 0.05, Fig. S3B). For data lengths that exceeded 0.2 s, all electrode configurations achieved classification accuracies above 95%, with no significant differences observed (P > 0.05). In the frequency–phase–space encoding BCI system with 80 to 200 targets, increasing electrode density consistently improved classification accuracies across all data lengths (P < 0.05). For example, in the 200-target BCI system, the classification accuracies for the 9/64, 21/64, 32/128, and 66/256 electrode configurations were 29.71% ± 2.65%, 43.13% ± 2.71%, 48.54% ± 2.68%, and 53.82% ± 2.87% at 0.1 s, and 70.81% ± 2.57%, 81.37% ± 1.80%, 84.27% ± 1.71%, and 87.04% ± 1.57% at 0.5 s, respectively. Notably, the performance improvements associated with increased electrode density were consistently observed across different decoding algorithms (Fig. S4).

Subsequently, the impact of electrode density on the actual ITR of the BCI system was evaluated. The actual ITR (measured in bits per minute) was calculated by considering the total time required for each target selection, which includes both stimulation time and gaze shift time, providing a more accurate reflection of the system’s true command transmission speed [1]. With a high-density electrode configuration of 66/256, the BCI system achieved peak actual ITR values of 432.27 bpm ± 5.51 bpm (data length: 0.2 s, accuracy: 97.58% ± 0.59%), 470.64 bpm ± 8.97 bpm (data length: 0.2 s, accuracy: 92.59% ± 1.06%), 465.06 ± 15.60 bpm (data length: 0.2 s, accuracy: 86.38% ± 1.90%), 461.69 bpm ± 18.19 bpm (data length: 0.2 s, accuracy: 82.26% ± 2.22%), and 416.22 ± 14.18 bpm (data length: 0.3 s, accuracy: 81.33% ± 1.94%) for target numbers of 40, 80, 120, 160, and 200, respectively (Fig. 2C). In comparison, the commonly used 9/64 electrode configuration in previous studies achieved peak actual ITR values of 372.53 bpm ± 8.57 bpm (data length: 0.3 s, accuracy: 96.64% ± 1.28%), 383.98 ± 11.56 bpm (data length: 0.3 s, accuracy: 88.59% ± 1.68%), 353.20 bpm ± 18.99 bpm (data length: 0.3 s, accuracy: 78.31% ± 2.87%), 337.12 bpm ± 15.61 bpm (data length: 0.4 s, accuracy: 78.69% ± 2.51%), and 289.64 ± 15.59 bpm (data length: 0.4 s, accuracy: 68.17% ± 2.63%) for the 40-, 80-, 120-, 160-, and 200-target conditions, respectively. The differences in actual ITR across varying electrode densities were similar to those observed in classification accuracy. As the number of targets increased from 40 to 200, the actual ITR increase relative to the 9/64 configuration widened from 23.96% (P = 3.48 × 10^−4^, Cohen’s *d*_z_ = 1.46, 95% CI [45.47 bpm, 100.82 bpm]), 35.78% (P = 3.17 × 10^−6^, Cohen’s *d*_z_ = 2.24, 95% CI [84.80 bpm, 140.52 bpm]), 56.93% (P = 1.25 × 10^−7^, Cohen’s *d*_z_ = 2.91, 95% CI [121.18 bpm, 178.15 bpm]), 68.04% (P = 6.93 × 10^−9^, Cohen’s *d*_z_ = 3.64, 95% CI [144.41 bpm, 196.24 bpm]) to 79.68% (P = 1.22 × 10^−9^, Cohen’s *d*_z_ = 4.15, 95% CI [145.11 bpm, 191.10 bpm]) for the 66/256 configuration (P < 0.001); widened from 22.37% (P = 4.33 × 10^−4^, Cohen’s *d*_z_ = 1.43, 95% CI [41.75 bpm, 94.42 bpm]), 31.28% (P = 7.78 × 10^−6^, Cohen’s *d*_z_ = 2.08, 95% CI [71.82 bpm, 124.11 bpm]), 48.05% (P = 6.46 × 10^−7^, Cohen’s *d*_z_ = 2.55, 95% CI [97.67 bpm, 151.80 bpm]), 55.58% (P = 1.81 × 10^−8^, Cohen’s *d*_z_ = 3.39, 95% CI [115.27 bpm, 160.42 bpm]) to 62.67% (P = 8.11 × 10^−10^, Cohen’s *d*_z_ = 4.28, 95% CI [114.85 bpm, 149.90 bpm]) for the 32/128 configuration (P < 0.001); widened from 19.32% (P = 2.89 × 10^−4^, Cohen’s *d*_z_ = 1.49, 95% CI [37.10 bpm, 80.94 bpm]), 23.85% (P = 8.34 × 10^−6^, Cohen’s *d*_z_ = 2.06, 95% CI [54.73 bpm, 94.90 bpm]), 36.52% (P = 2.35 × 10^−7^, Cohen’s *d*_z_ = 2.77, 95% CI [76.46 bpm, 114.70 bpm]), 40.96% (P = 7.93 × 10^−9^, Cohen’s *d*_z_ = 3.60, 95% CI [86.16 bpm, 117.47 bpm]) to 45.50% (P = 8.62 × 10^−10^, Cohen’s *d*_z_ = 4.26, 95% CI [82.92 bpm, 107.71 bpm]) for the 21/64 configuration (Fig. S3C, data length: 0.2 s).

When the visual information transfer pathway was considered as a channel from a communication systems perspective, no stimulation was received during gaze shifts. Therefore, the theoretical ITR (measured in bits per second, bps) was further calculated by considering only the stimulation time under ultra-short window lengths, thereby representing the theoretical communication limits of the current BCI systems [1]. With a data length of 0.04 s and an electrode configuration of 66/256, the BCI systems achieved peak theoretical ITR values of 61.13 bps ± 5.56 bps, 58.45 bps ± 5.31 bps, 49.69 bps ± 5.08 bps, 45.71 bps ± 4.80 bps, and 38.45 bps ± 3.92 bps for target numbers of 40, 80, 120, 160, and 200, respectively (Fig. 2D). As the number of targets increased from 40 to 200, the theoretical ITR increase relative to the 9/64 configuration widened from 83.66% (P = 6.54 × 10^−8^, Cohen’s *d*_z_ = 3.06, 95% CI [20.89 bps, 30.11 bps]), 134.02% (P = 5.41 × 10^−9^, Cohen’s *d*_z_ = 3.71, 95% CI [26.00 bps, 35.12 bps]), 152.66% (P = 2.14 × 10^−8^, Cohen’s *d*_z_ = 3.34, 95% CI [23.14 bps, 32.34 bps]), 177.95% (P = 3.26 × 10^−8^, Cohen’s *d*_z_ = 3.23, 95% CI [22.46 bps, 31.75 bps]) to 195.56% (P = 3.13 × 10^−8^, Cohen’s *d*_z_ = 3.24, 95% CI [19.55 bps, 27.61 bps]) for the 66/256 configuration (P < 0.001); widened from 79.99% (P = 2.53 × 10^−8^, Cohen’s *d*_z_ = 3.30, 95% CI [19.50 bps, 27.38 bps]), 112.87% (P = 1.34 × 10^−8^, Cohen’s *d*_z_ = 3.46, 95% CI [21.36 bps, 29.50 bps]), 124.02% (P = 2.93 × 10^−8^, Cohen’s *d*_z_ = 3.26, 95% CI [18.15 bps, 25.59 bps]), 141.24% (P = 2.58 × 10^−8^, Cohen’s *d*_z_ = 3.29, 95% CI [17.18 bps, 24.13 bps]) to 153.08% (P = 5.13 × 10^−8^, Cohen’s *d*_z_ = 3.12, 95% CI [14.74 bps, 21.10 bps]) for the 32/128 configuration (P < 0.001); widened from 55.50% (P = 2.15 × 10^−7^, Cohen’s *d*_z_ = 2.79, 95% CI [13.84 bps, 20.70 bps]), 77.68% (P = 2.08 × 10^−7^, Cohen’s *d*_z_ = 2.80, 95% CI [14.66 bps, 21.90 bps]), 84.27% (P = 4.56 × 10^−7^, Cohen’s *d*_z_ = 2.62, 95% CI [12.46 bps, 19.13 bps]), 93.97% (P = 4.65 × 10^−7^, Cohen’s *d*_z_ = 2.62, 95% CI [11.52 bps, 17.69 bps]) to 103.07% (P = 1.19 × 10^−6^, Cohen’s *d*_z_ = 2.43, 95% CI [9.68 bps, 15.39 bps]) for the 21/64 configuration (P < 0.001, Fig. S3D, data length: 0.04 s).

**Fig. 3.**
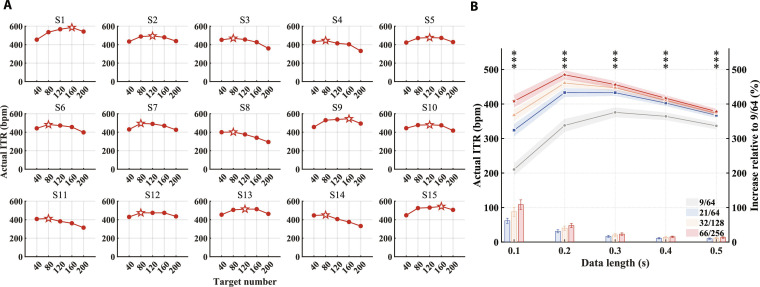
Personalizing the BCI system parameters for each subject further enhances the actual ITR. (A) The relationship between the actual ITR and target number for each subject, with hollow star symbols representing the peak values. (B) The average actual ITR (lines) after personalizing the system parameters for 15 subjects, along with the actual ITR increase relative to the 9/64 configuration (bars). The asterisks indicate the significance level of the 4 electrode configurations, calculated using the one-way repeated-measures analysis of variance (RMANOVA) (***P < 0.001). The shaded area and error bars represent the standard errors (number of participants = 15).

The average results across subjects indicated that the actual ITR increased as the target number rose from 40 to 80, but then declined consistently as the target number further increased from 80 to 200. However, the relationship between target number and actual ITR varied across individual subjects (Fig. 3A). To maximize system performance, the optimal target number and fixation combination for each subject were selected based on the highest actual ITR value (Table S1). A total of 0, 8, 4, 3, and 0 subjects achieved peak actual ITR at target numbers of 40, 80, 120, 160, and 200, respectively. Among the 15 subjects, the highest actual ITR was 586.10 bpm (data length: 0.2 s, accuracy: 96.42%) with 160 targets, while the lowest actual ITR was 401.78 bpm (data length: 0.2 s, accuracy: 84.10%) with 80 targets. Fig. 3B shows the average actual ITR across 15 subjects after personalizing the system parameters. When the data length was 0.2 s, the actual ITR for the 66/256 configuration reached a peak of 484.76 ± 12.68 bpm, which was significantly higher than that of the 32/128 (0.2 s, 460.75 bpm ± 12.32 bpm, P = 1.69 × 10^−4^, Cohen’s *d*_z_ = 1.57, 95% CI [15.54 bpm, 32.47 bpm]), 21/64 (0.2 s, 433.14 bpm ± 12.93 bpm, P = 1.14 × 10^−6^, Cohen’s *d*_z_ = 2.44, 95% CI [39.89 bpm, 63.35 bpm]), and 9/64 (0.3 s, 375.78 bpm ± 14.15 bpm, P = 3.44 × 10^−6^, Cohen’s *d*_z_ = 2.22, 95% CI [60.52 bpm, 100.65 bpm], Fig. S5) configurations.

#### Online BCI performance

To further validate the performance of the high-density EEG-based BCI system, an online experiment was conducted. Once the subjects completed a target selection, real-time classification results were provided to them in the form of visual feedback. It was important to note that, although the actual ITR in the offline experiment peaked when the data length was 0.2 s, not all subjects were able to switch their visual attention quickly enough to meet this standard. Therefore, in addition to the target number and fixation combinations optimized in the offline experiment, the online experiment further selected an appropriate stimulus duration for each subject. The interstimulus interval was uniformly set to 0.5 s for all subjects to cue the location of the next target. The online experiment successfully recalled 10 subjects who had previously participated in the offline experiment. Table 2 lists the system parameters and performance for each subject. The average online actual ITR reached 472.72 bpm ± 15.06 bpm.

**Table 2.** System parameters and the corresponding performance achieved in the online BCI experiment for each subject

Subject	Target number	Fixation combination	Stimulation time/s	Accuracy /%	Theoretical ITR/bps	Actual ITR /bpm
S1	160	Right, down, left, up	0.25	96.88	27.57	551.42
S3	80	Right, left	0.2	93.75	27.95	479.20
S4	80	Down, up	0.25	91.00	21.27	425.45
S5	120	Right, left, up	0.2	88.67	28.08	481.36
S7	80	Down, up	0.2	95.25	28.73	492.58
S10	120	Right, left, up	0.2	82.67	25.23	432.58
S11	80	Down, up	0.2	87.50	24.95	427.75
S12	80	Right, left	0.2	91.75	26.95	462.08
S14	80	Down, up	0.3	94.75	18.98	427.05
S15	160	Right, down, left, up	0.25	96.50	27.39	547.77
Mean	–	–	–	–	25.71	472.72
STE	–	–	–	–	1.02	15.06

STE, standard error

### Principles of spatial information decoding

**Fig. 4.**
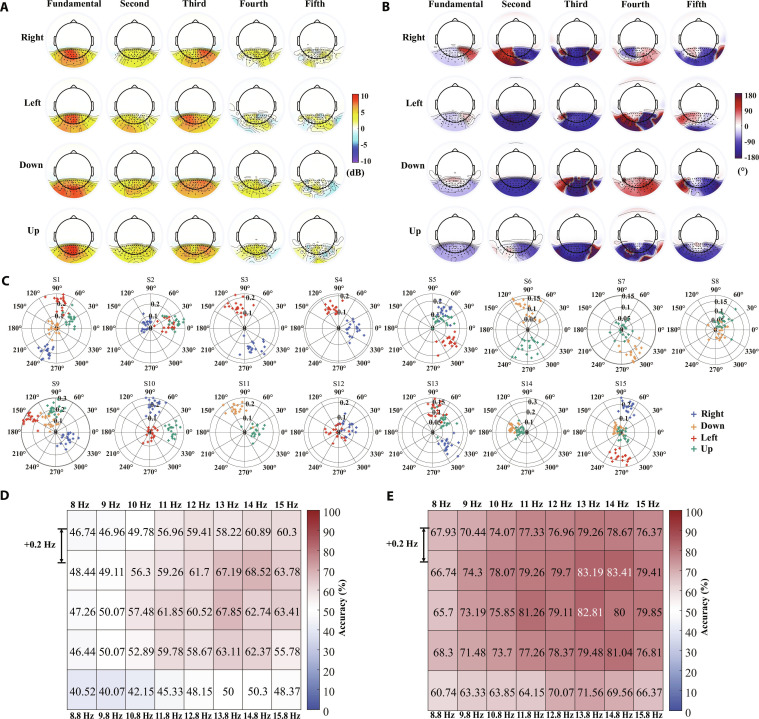
Spatial response patterns and spatial information decoding performance. (A) The average SNR topographies at the fundamental and harmonic frequencies across 15 subjects for the 14-Hz flicker stimulus (see Fig. S6 for additional details). (B) The phase topographies at the fundamental and harmonic frequencies for a representative subject in response to the 14-Hz flicker stimulus (see Fig. S7 for additional details). (C) Complex spectrum features after task-discriminant component analysis (TDCA) spatiotemporal filtering, with each subject presenting only the fixation points selected through personalized customization. Each cross represents an individual trial, with different colors corresponding to different fixation points. (D) Classification accuracy for 5 fixation points on each of the 40 flickers using the spatiotemporal filter trained on the 5-target classification task (data length: 0.2 s). (E) Classification accuracy for 5 fixation points on each of the 40 flickers using the spatiotemporal filter trained on the 200-target classification task (data length: 0.2 s).

The retinal-to-visual cortex mapping suggests that when visual input appears at different locations within the visual field, different regions of the visual cortex are activated [40,41]. Meanwhile, previous SSVEP studies have shown that stimulation frequencies within the low-frequency range tend to elicit highly similar spatial topographies [2,8]. Motivated by these findings, the present study primarily focuses on how different fixation points modulate the spatial distribution of SSVEP responses, using high-density EEG to characterize these spatial differences. Fig. 4A shows the distribution of mean SNR at the fundamental and harmonic frequencies across 15 subjects (see Fig. S6 for additional details). Overall, when the fixation point was on the right, stimuli presented in the left visual field elicited a clear rightward lateralization of the response distribution at the harmonic frequencies. Conversely, when the fixation point was on the left, the lateralization was observed in the opposite direction. When the fixation point was located at the upper or lower visual field, more pronounced differences in response distribution were observed at the fundamental frequency. Specifically, with a down fixation point, the response distribution was broader and stronger near the CBz electrode, while with an up fixation point, the response distribution was more concentrated and stronger near the Pz electrode. The response phase topography for a representative subject (S1) is shown in Fig. 4B (see Fig. S7 for other subjects). The phase distribution at both the fundamental and harmonic frequencies also exhibited specificity based on the fixation point location. To more intuitively describe the aforementioned differences for various fixation points, the TDCA spatiotemporal filter was applied for dimensionality reduction of high-density EEG activity [8], and the low-dimensional features were subsequently visualized in the form of complex spectrum (Fig. 4C). The number and combination of fixation points were personalized based on each individual’s actual ITR, with clear separation observed among different fixation points. Subsequently, the spatial information decoding ability under high-density EEG was further quantified. A 5-fixation-point classification task was performed for each flicker. With a data length of 0.2 s, the average classification accuracy across 40 flickers was 54.97% ± 1.23%. Fig. 4D shows that flickers located at the center of the interface exhibited higher classification accuracy than those at the edges, which may be due to the former’s additional contribution from peripheral flickers. To further evaluate the spatial information decoding capability in the 200-target classification task, the TDCA spatiotemporal filter trained on the 200-class task was applied to the 5-class task for each flicker. The average classification accuracy across 40 flickers increased to 74.47% ± 0.98% (Fig. 4E).

### High-density EEG provides richer neural activity information

**Fig. 5.**
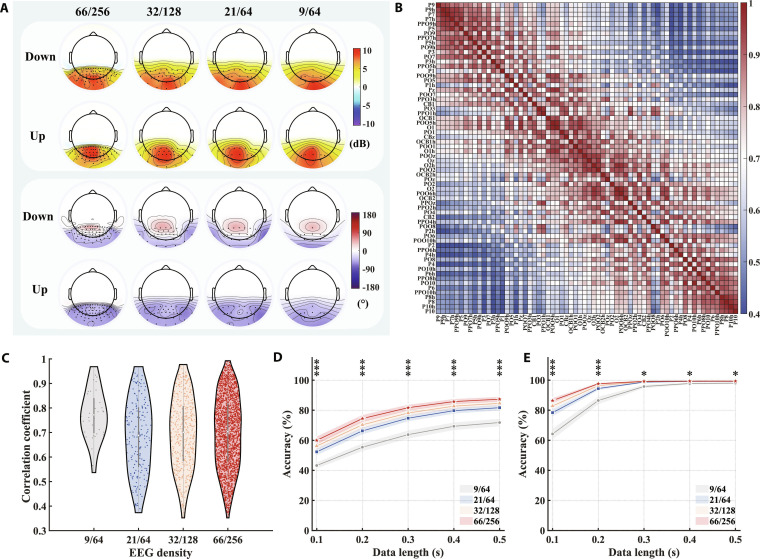
High-density EEG enables finer capture of response features and more precise decoding of visual information. (A) Grand-average SNR topographies (across 15 subjects) and phase topographies for a representative subject at the fundamental frequency in response to the 14-Hz flicker stimulus under the 9/64, 21/64, 32/128, and 66/256 configurations. (B) Pearson correlation coefficients between all pairs of 66 electrodes under the 66/256 configurations. (C) A violin plot of the distribution of correlation coefficients under the 9/64, 21/64, 32/128, and 66/256 electrode configurations. (D) Accuracy for 5-fixation-point classification using the spatiotemporal filter trained on the 200-target classification task. (E) Accuracy for 40-flicker classification using the spatiotemporal filter trained on the 200-target classification task. The asterisks indicate the significance level of the 4 electrode configurations, calculated using the one-way RMANOVA. *P < 0.05 and ***P < 0.001. The shaded area represents the standard errors (number of participants = 15).

To provide a more explicit comparison of the impact of electrode density on spatial response patterns, response intensity and phase topographies for 4 electrode configurations are depicted in Fig. 5A. As electrode density decreases, the contour lines in the topographies gradually smooth out, and the detailed variations in response intensity and phase in the parieto-occipital region become increasingly less discernible. Fig. 5B displays the correlation coefficients between all pairs of 66 electrodes in the parieto-occipital region. The correlation decreased as the interelectrode distance increased. Fig. 5C shows the distribution of correlation coefficients for the 4 electrode configurations. Due to its smaller coverage area compared to the other 3 configurations (21/64: 0.68, 32/128: 0.70, and 66/256: 0.70), the 9/64 configuration exhibited the highest mean correlation coefficient of 0.77. The number of electrode pairs with correlation coefficients lower than 0.6 for the 9/64, 21/64, 32/128, and 66/256 configurations were 2, 68, 140, and 610, respectively. To compare the impact of electrode density on spatial information decoding and frequency information decoding, the same spatiotemporal filter (trained on the 200-class task) was applied to process the SSVEP responses of 5 fixation points and 40 flickers, followed by classification (Fig. 5D and E). As assessed by 2-way (density × data length) RMANOVA, the interaction between electrode density and data length was statistically significant for both the 5-fixation-point classification [*F*(1.52, 21.25) = 3.89, P = 0.046, partial *η*^2^ = 0.22] and the 40-flicker classification [*F*(1.47, 20.57) = 82.89, P = 1.02 × 10^−9^, partial *η*^2^ = 0.86]. The enhancement in spatial information decoding capability remained significant across all data lengths as electrode density increased (Fig. S8). When the data length was 0.1 s, the 66/256 configuration outperformed the 9/64, 21/64, and 32/128 configurations by 16.73% (P = 1.91 × 10^−9^, Cohen’s *d*_z_ = 4.01, 95% CI [14.42%, 19.04%]), 7.65% (P=5.67 × 10^−8^, Cohen’s *d*_z_ = 3.10, 95% CI [6.28%, 9.02%]), and 3.76% (P = 3.55 × 10^−4^, Cohen’s *d*_z_ = 1.46, 95% CI [2.34%, 5.19%]), respectively. When the data length was 0.5 s, the 66/256 configuration showed improvements of 15.53% (P = 7.06 × 10^−8^, Cohen’s *d*_z_ = 3.04, 95% CI [12.70%, 18.35%]), 5.65% (P=1.29 × 10^−7^, Cohen’s *d*_z_ = 2.90, 95% CI [4.57%, 6.73%]), and 2.76% (P=2.69 × 10^−6^, Cohen’s *d*_z_ = 2.27, 95% CI [2.09%, 3.44%]) over the 9/64, 21/64, and 32/128 configurations, respectively. In contrast, with regard to frequency information decoding capability, no significant differences were observed among most electrode configurations once a certain data length was reached. Specifically, when the data length was 0.1 s, the 66/256 configuration outperformed the 9/64, 21/64, and 32/128 configurations by 22.19% (P = 2.97 × 10^−7^, Cohen’s *d*_z_ = 2.72, 95% CI [17.67%, 26.71%]), 8.01% (P = 1.01 × 10^−5^, Cohen’s *d*_z_ = 2.03, 95% CI [5.83%, 10.20%]), and 3.58% (P = 1.13 × 10^−5^, Cohen’s *d*_z_ = 2.01, 95% CI [2.59%, 4.57%]), respectively. When the data length was 0.5 s, the 66/256 configuration showed improvements of 1.32% (P=0.04, Cohen’s *d*_z_ = 0.82, 95% CI [0.43%, 2.21%]), 0.12% (P=0.28, Cohen’s *d*_z_ = 0.56, 95% CI [0.002%, 0.23%]), and 0.04% (P=1, Cohen’s *d*_z_ = 0.38, 95% CI [−0.02%, 0.10%]) over the 9/64, 21/64, and 32/128 configurations, respectively.

**Fig. 6.**
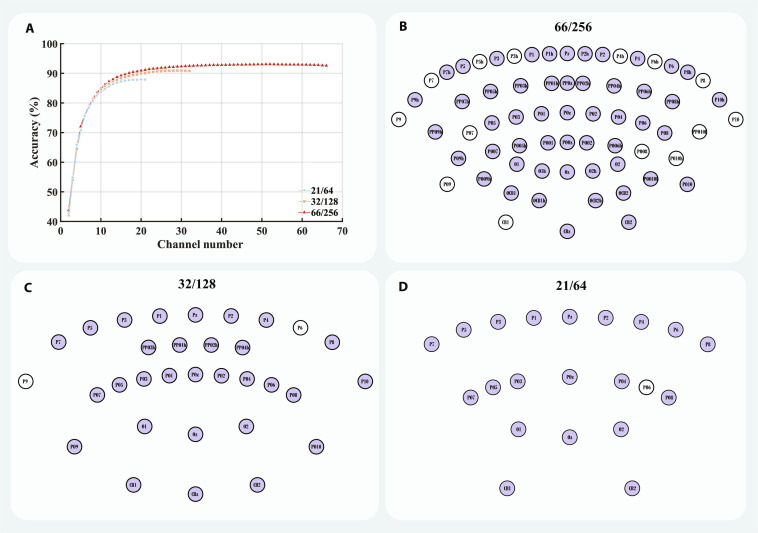
Optimization of electrode number and combinations under different electrode densities. (A) Classification accuracy achieved by the optimal electrode combinations at different electrode numbers in an 80-target classification task with a data length of 0.2 s. (B) The optimal electrode layout for the optimal electrode number of 52 under the 66/256 configuration. (C) The optimal electrode layout for the optimal electrode number of 30 under the 32/128 configuration. (D) The optimal electrode layout for the optimal electrode number of 20 under the 21/64 configuration. Purple represents the selected electrodes, while white represents the unselected electrodes.

The greedy algorithm was used to search for the optimal number of electrodes and the corresponding electrode combinations. This method served to evaluate potential redundancy in the current electrode configuration and to compare the performance differences resulting from varying electrode densities at the same electrode number. Given that more than half of the 15 subjects achieved the highest actual ITR with 80 targets, optimization was conducted in the 80-target classification task. In the 66/256 configuration, the accuracy peaked at 52 electrodes, showing a significant difference compared to the performance with 66 electrodes (93.07% vs 92.59%, P = 2.8 × 10^−3^, Cohen’s *d*_z_ = 0.94, 95% CI [0.20%, 0.77%], Fig. 6A). The corresponding optimal electrode combination is shown in Fig. 6B, with the unselected electrodes distributed in the peripheral areas. In the 32/128 and 21/64 configurations, the accuracy peaked at 30 and 20 electrodes, respectively, which almost included all electrodes from the respective configurations (Fig. 6C and D). No significant performance differences were observed compared to using all electrodes (32/128: 90.94% vs. 90.82%, P = 0.08, Cohen’s *d*_z_ = 0.48, 95% CI [−0.02%, 0.25%], 21/64: 87.94% vs. 87.88%, P = 0.51, Cohen’s *d*_z_ = 0.17, 95% CI [−0.13%, 0.25%]). When the number of electrodes was fewer than 10, electrode density had no significant effect on the performance of the optimal electrode combinations (P > 0.05; Fig. S9). However, when the number of electrodes ranged from 11 to 21, the performance of the optimal electrode combinations searched under the 66/256 configuration was significantly better than that of the 21/64 configuration with the same number of electrodes (P < 0.05). Similarly, when the number of electrodes ranged from 22 to 32, the performance of the optimal electrode combinations searched under the 66/256 configuration was significantly better than that of the 32/128 configuration with the same number of electrodes (P < 0.05).

### Dynamic window classification algorithm further boosts ITR

**Fig. 7.**
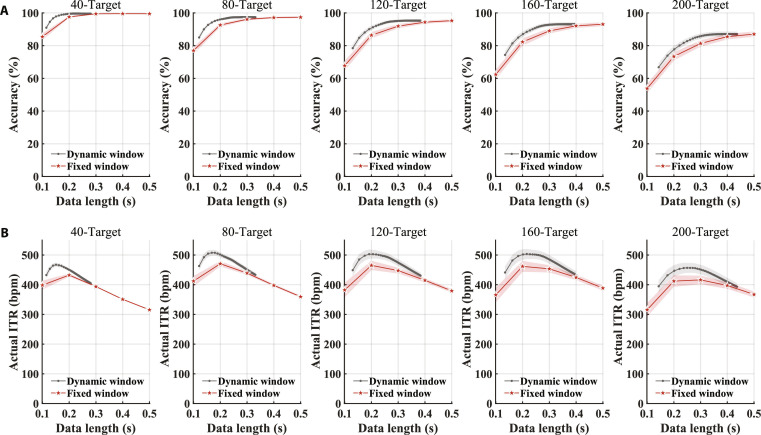
Dynamic window classification algorithm further enhances the performance of the BCI system. (A) Comparison of classification accuracy using fixed and dynamic window classification algorithms. (B) Comparison of actual ITR using fixed and dynamic window classification algorithms. The fixed window algorithm output classification results at 5 fixed data lengths, ranging from 0.1 to 0.5 s in 0.1-s increments (indicated by the red stars). The dynamic window algorithm determined the data length for output based on whether the risk cost fell below the threshold. The gray dots represent the average classification duration across trials at different thresholds, along with the corresponding classification performance. The shaded area represents the standard errors (number of participants = 15).

Considering the variability in spatial information decoding capabilities across subjects, a dynamic-window classification algorithm [9] was adopted to further enhance system performance. In this framework, stricter decision thresholds lead to longer output times. Fig. 7 shows the average output time across all trials and the corresponding classification performance for different thresholds. As the number of targets increased from 40 to 200, the average output time required to achieve peak actual ITR using the dynamic window classification algorithm also increased (40-target: 0.151 s, 97.82% ± 0.34%, 80-target: 0.168 s, 94.16% ± 0.76%, 120-target: 0.192 s, 90.08% ± 1.37%, 160-target: 0.216 s, 88.45% ± 1.57%, 200-target: 0.250 s, 82.77% ± 1.75%). Furthermore, compared to the fixed window method, the dynamic window classification algorithm demonstrated a significant improvement in peak actual ITR (40-target: 432.27 bpm ± 5.51 bpm vs. 467.28 bpm ± 6.69 bpm, P = 5.58 × 10^−10^, Cohen’s *d*_z_ = 3.85, 95% CI [29.97 bpm, 40.05 bpm], 80-target: 470.64 bpm ± 8.97 bpm vs. 507.59 bpm ± 9.93 bpm, P = 1.87 × 10^−11^, Cohen’s *d*_z_ = 4.96, 95% CI [32.82 bpm, 41.08 bpm], 120-target: 465.06 ± 15.60 bpm vs. 502.89 bpm ± 15.64 bpm, P = 5.20 × 10^−13^, Cohen’s *d*_z_ = 6.45, 95% CI [34.58 bpm, 41.08 bpm], 160-target: 461.69 bpm ± 18.19 bpm vs. 503.48 bpm ± 16.92 bpm, P = 2.11 × 10^−9^, Cohen’s *d*_z_ = 3.48, 95% CI [35.13 bpm, 48.44 bpm], 200-target: 416.22 bpm ± 14.18 bpm vs. 456.72 bpm ± 16.87 bpm, P = 1.92 × 10^−9^, Cohen’s *d*_z_ = 3.50, 95% CI [34.09 bpm, 46.90 bpm]).

## Discussion

**Fig. 8.**
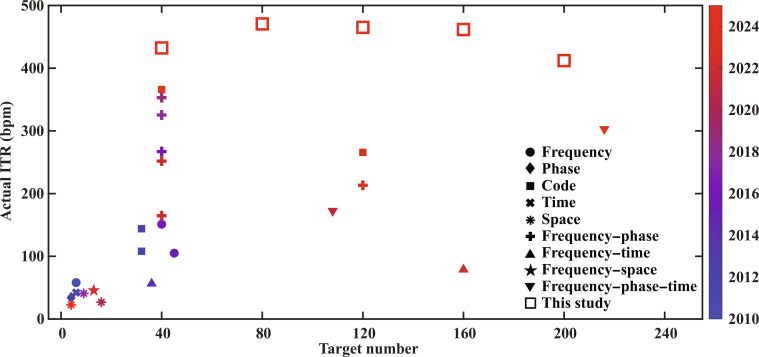
Comparison of encoding methods, target number, and actual ITR of visual BCI systems in recent years with the present study [1–23]. The detailed parameters used to compute the actual ITR in each study are provided in Table S2.

Previous neurophysiological studies using ultra-dense scalp electrode arrays have shown that reducing interelectrode distance improves the SNR of VEPs and reveals finer spatial patterns in the early visual cortex [28,37,38,46]. However, these works did not address whether, and under which conditions, such gains in spatial resolution could translate into higher communication speed in practical BCI systems, nor did they answer how dense an EEG montage needs to be to satisfy specific decoding demands. In contrast, most state-of-the-art visual BCIs still rely on 64-channel caps and primarily exploit temporal encoding schemes. In addition, although previous studies have demonstrated that differences in the relative positions between fixation points and visual stimuli can be exploited to discriminate targets, spatial-only encoding is typically constrained by a limited number of targets, relatively large stimulus patches, and low system ITR [10,13,15]. Building on this gap, the present study designed a hybrid frequency–phase–space encoding paradigm, aimed at investigating the advantages of high-density EEG from both frequency–phase information decoding and spatial information decoding perspectives. Under the 66/256 high-density electrode configuration, the constructed BCI system achieved a relatively high offline actual ITR, with a peak of 470.64 bpm ± 8.97 bpm (data length: 0.2 s, accuracy: 92.59% ± 1.06%). After personalizing the system parameters of target number, fixation combinations, and stimulus duration for individual participants, an online actual ITR of 472.72 bpm ± 15.06 bpm was achieved (Table 2), fully validating the reliability and robustness of the system’s performance. The proposed SSVEP-BCI systems offer the following notable advantages:

1. Efficient integration of spatiotemporal information for large command set encoding. The number of targets that can be encoded using frequency encoding method is limited by the dominant frequency bands of the VEP response [17], while the space encoding method is constrained by the number of spatial positions capable of eliciting robust responses [10,15]. The hybrid frequency–phase–space encoding method proposed in this study addressed these limitations by assigning frequency and phase parameters to multiple flickers and placing multiple fixation points within each flicker. This approach increased the command set size from 40 to 200, achieving a leading number of targets among reported BCI systems [1–23].

2. High-resolution spatiotemporal decoding with compact visual stimuli. Traditional BCI systems typically required larger display screens to accommodate an expanded command set, which increased the difficulty for users in locating target positions [6,7,11]. By integrating hybrid spatiotemporal encoding method with the high-density EEG decoding strategy, this study enhanced the visual resolution of spatiotemporal information decoding. Compared to the stimulus size exceeding 3° used in the classic 40-target BCI system [1–3,8,33], the average stimulus size in the 80-, 120-, 160-, and 200-target systems were reduced to 2.35°, 1.92°, 1.66°, and 1.49°, thereby improving the user experience when interacting with large command set systems.

3. Breakthrough in ITR. Fig. 8 compares the encoding methods, number of targets, and actual ITRs of recently reported visual BCI systems with those of the present study [1–23]. All actual ITR values shown in this figure were calculated by taking into account both the stimulus duration and the gaze-shift interval. The introduction of the frequency–phase encoding method and the TRCA decoding strategy in 2018 marked a substantial advance in communication speed. In that study, a 40-target SSVEP-BCI system was developed on an interface that was nearly identical in size to that used in the present work and achieved an actual ITR of 325.33 bpm, enabling one target selection every 0.8 s (stimulus duration: 0.3 s, interstimulus interval: 0.5 s, accuracy: 89.83%, 12 training blocks) [2]. By 2024, another 40-target BCI system utilizing white noise sequence encoding method achieved the highest reported actual ITR of 366.05 bpm (stimulus duration: 0.2 to 0.3 s, interstimulus interval: 0.5 s, accuracy: 95%) [1]. Owing to the expanded command set and the contribution of high-density EEG to feature extraction, this study achieved another improvement in actual ITR, reaching 472.72 bpm ± 15.06 bpm. It is worth mentioning that even the 9/64 configuration, which was commonly used in previous studies, achieved an actual ITR breakthrough of 383.98 bpm ± 11.56 bpm with 80 targets.

The optimal electrode density required to meet the demands of visual BCI systems is a key issue that has garnered substantial attention in both research and application fields, yet remains challenging to answer [28,47]. In this study, this issue required a comprehensive consideration of the encoding method, target number, and data length to draw a conclusion. In the 40-target system using frequency–phase encoding, both an increase in electrode coverage (from the 9/64 to the 21/64 configuration) and an increase in electrode density (from the 21/64 to the 66/256 configuration) resulted in significant improvements in classification performance when the data length was less than or equal to 0.2 s. However, when the data length reached 0.3 s, these improvements became negligible, and a 21/64 electrode density was sufficient. In contrast, in BCI systems with hybrid frequency–phase–space encoding method, significant performance differences among the 4 electrode configurations were observed across all target numbers (from 80 to 200 targets) and data lengths (from 0.1 to 0.5 s). Furthermore, as the target number increased, the performance gap between different electrode configurations became more pronounced (Fig. 2). Given that this study focuses on basic visual information, such as periodic brightness changes and the relative position of the fixation point and visual stimuli, it can be inferred that as the difficulty of the decoding task (e.g., increased target numbers) or the complexity of the visual stimuli (e.g., natural image stimuli) increases, the demand for higher electrode density will also rise.

In addition, we performed electrode subset optimization for the 80-target classification task, in which the peak actual ITR was obtained. The electrode–performance curve exhibited a clear pattern of diminishing returns and reached its maximum at 52 channels (Fig. 6A and B), suggesting that some peripheral channels in the high-density configuration may be redundant for this moderately difficult classification task. When the optimized 52/256 configuration was compared with the full 66/256 configuration, the 52/256 configuration achieved a slightly higher mean performance in the 40- to 80-target conditions, whereas the full 66/256 configuration showed a numerically higher performance in the 120- to 200-target conditions (Fig. S10A). Electrode subset optimization for the 200-target classification task (Fig. S11) peaked at 60 electrodes, suggesting that the optimal channel count increases for more complex tasks. As shown in Fig. 6B and Fig. S11, the discarded electrodes were primarily located at the periphery of the 66-channel montage, implying a limited impact on electrode density. Furthermore, SNR and interchannel correlation analyses revealed that the discarded channels were mainly low-SNR peripheral electrodes and exhibited weaker correlations with the remaining channels (Fig. S10B and C), which may be consistent with the possibility that some peripheral electrodes contribute little to simpler decoding tasks but provide complementary spatial information under more complex conditions.

As the electrode density increased, subtle variations in the response characteristics across electrodes could be captured (Fig. 5A), which facilitated the enhancement of visual information decoding performance. A comprehensive comparison was made between the contributions of high-density EEG to both frequency information and spatial information decoding. Previous studies have shown that SSVEPs induced by different frequencies exhibit similar spatial distributions, concentrated in the parieto-occipital regions without any lateralization [2]. However, differences in the relative spatial positions between the fixation point and visual stimuli could lead to varying degrees and directions of lateralized amplitude distributions, as well as differentiated phase distributions in the induced SSVEPs (Fig. 4). This explained the principle underlying the distinguishability of fixation points within the same flicker under identical temporal parameters (frequency and phase values) and elucidated why increasing electrode density results in more significant improvements in spatial information decoding tasks compared to frequency information decoding (Fig. 5D and E).

In spatial information decoding tasks, significant performance differences existed among different fixation combinations (Fig. 2A). Interestingly, the fixation combination that yielded the best classification performance corresponded to those with the farthest physical distance. Beyond the choice of fixation combinations, the absolute placement of flickers within the 5 × 8 array also influenced spatial decoding performance (Fig. 4D and E). Zhang et al. [48] reported that SSVEP amplitude decreases steeply with eccentricity between 0° and 4°, and more gradually between 6° and 10° of visual angle. In the present interface, each flicker subtended approximately 3.3° (144 pixels), with a gap of about 1.2° (50 pixels) between adjacent flickers. Consequently, items near the center of the array had more neighboring flickers within a relatively small eccentricity range than items at the edges. Consistent with this geometry, Fig. 4D and E show that centrally located flickers exhibit higher 5-fixation-point decoding accuracy, suggesting that neighboring patches with distinct frequency–phase assignments enhance spatial discriminability by providing additional, position-specific SSVEP components.

Despite the promising performance demonstrated in this work, several limitations should be considered when interpreting the findings. All measurements were obtained under controlled laboratory conditions with stabilized head position, fixed viewing distance, and constant ambient illumination. Therefore, the reported ITRs should be interpreted as upper bounds on the communication speed that can be achieved with this paradigm. In more naturalistic environments, the impact of motion- and blink-related artifacts is likely stronger. Consequently, performance is expected to decrease, especially for very short decision windows. Quantifying this degradation will be an important direction for future work. Possible approaches include comparing sessions with and without head stabilization, explicitly introducing instructed head movements and blinks, and developing dedicated artifact-robust decoding strategies. In addition, the absence of dedicated electrooculography and eye-tracking recordings prevents a direct assessment of how eye movements and minor fixation deviations contribute to the observed spatial decoding performance. Future studies will therefore incorporate these measurements to disentangle the contribution of neural activity from eye movement artifacts. A further limitation is that all experiments were conducted in a relatively homogeneous group of healthy young adults with normal or corrected-to-normal vision and no known neurological disorders. Therefore, the current results cannot be directly generalized to older adults or to users with visual or mild neurological impairments, whose visually evoked potentials, visual acuity, and attentional control may differ from those of our sample. Future research should evaluate the robustness and usability of the proposed frequency–phase–space encoding paradigm and high-density EEG decoding strategy in more diverse populations, including individuals with common refractive errors, age-related changes in vision, or mild neurological conditions, and, if necessary, adapt stimulus parameters (e.g., contrast, size, and luminance) to their specific visual capabilities. Such studies will be essential for assessing the practical utility of the system in real-world assistive and rehabilitation scenarios. From a practical standpoint, the current high-density implementation used 66 parieto-occipital electrodes of a 256-channel cap, with a preparation time of approximately 30 to 50 min per session and subjective comfort comparable to conventional whole-head 64-channel gel-based systems. Nevertheless, dense montages require more hardware channels and maintenance than low-density systems, which are cheaper and easier to deploy but sacrifice part of the performance gains observed here. In this study, electrode density was manipulated by deriving the 32/128, 21/64, and 9/64 configurations from the 66/256 configuration. Although these reduced-density configurations were constructed by selecting electrodes that match the layouts of 128-channel and 64-channel Quik-Cap Neo Net caps, subtle differences between these configurations and recordings obtained with dedicated low-density caps cannot be fully excluded. Future work will therefore include experiments that directly compare configurations derived from high-density recordings with data obtained using low-density caps under otherwise identical conditions, and systematically evaluate caps of different densities in terms of setup time, maintenance requirements, user comfort, and decoding performance. Overall, in the present context, high-density EEG should be viewed primarily as a scientific tool for elucidating how electrode density shapes decoding performance across task types and difficulty levels, with the resulting design principles intended to inform more practical, application-specific montages for future engineering and product development.

Beyond these practical considerations, there is substantial scope to further advance the spatial encoding and decoding framework introduced here. Future research could increase the number of fixation points and employ microneedle electrode arrays with millimeter-level spatial resolution [49] to precisely map spatial locations to ultrahigh-density EEG response features. In addition to high-density acquisition technology, EEG source imaging methods offer another effective approach for enhancing spatial resolution by employing forward problem modeling and inverse problem solving to accurately project EEG data onto cortical voxels. Furthermore, beyond spatial locations, more complex spatial features such as color, shape, brightness, and contrast should also be taken into account. Developing predictive models that link visual stimulus spatial attributes with EEG responses could enable a transformative leap in spatiotemporal information encoding and decoding—from simple brightness modulation stimuli to sophisticated patterned stimuli, and ultimately to natural image stimuli. Ultimately, such advancements will drive the evolution of visual BCI technology from efficient interaction toward truly natural interaction.

## Conclusion

This study proposed a hybrid frequency–phase–space encoding paradigm combined with high-density EEG to build high-speed visual BCIs. A compact large-command-set scheme with 40 to 200 targets was generated by assigning 1 to 5 fixation points to each of the 40 flickers. Under the 66/256 electrode configuration, the offline system achieved a peak actual ITR of 470.64 bpm ± 8.97 bpm for 80 targets with an accuracy of 92.59% ± 1.06%, and the online system reached 472.72 bpm ± 15.06 bpm with customized fixation point combinations. Relative to the conventional 9/64 montage, the 66/256 configuration increased actual ITR by 23.96% to 79.68% as the task scaled from 40 to 200 targets. These findings indicate that high-density EEG is particularly beneficial for more difficult decoding tasks. Analyses of the 5-fixation-point and 40-flicker subtasks further showed that spatial information decoding has higher electrode density requirements than temporal information decoding, with performance gains of 15.53% versus 1.32% at a data length of 0.5 s. Together, these results show that a hybrid frequency–phase–space encoding strategy with high-density EEG enables high-speed, large-command-set visual BCIs and offers quantitative guidance for paradigm design and electrode configuration in future visual BCI studies.

## Data Availability

The raw EEG datasets from both the offline (15 subjects) and online (10 subjects) experiments are available at https://figshare.com/s/c962d29d7752e27176be. Any additional requests for information can be directed to, and will be fulfilled by, the corresponding authors.
