# Supplementary material for: A High-Speed Visual BCI Based on Hybrid Frequency–Phase–Space Encoding and High-Density EEG Decoding
Source: Cyborg Bionic Syst. 2026 Mar 26;7:0555. doi: 10.34133/cbsystems.0555 (PMC13018654; doi:10.34133/cbsystems.0555)
Supplement: Supplementary 1 — Figs. S1 to S11 Tables S1 and S2 Movie S1 [file cbsystems.0555.f1.zip › Fig.S1.pdf]

A

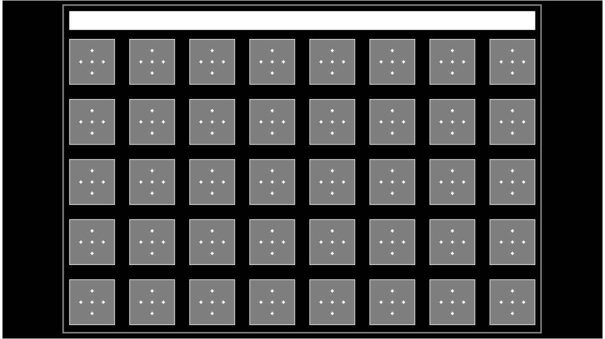

B

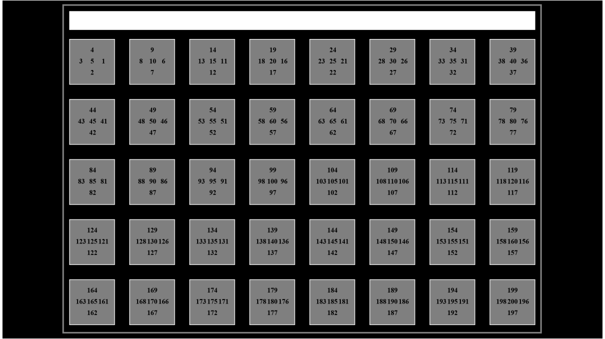
4 3 5 1 2
9 8 10 6 7
14 13 15 11 12
19 18 20 16 17
24 23 25 21 22
29 28 30 26 27
34 33 35 31 32
39 38 40 36 37
44 43 45 41 42
49 48 50 46 47
54 53 55 51 52
59 58 60 56 57
64 63 65 61 62
69 68 70 66 67
74 73 75 71 72
79 78 80 76 77
84 83 85 81 82
89 88 90 86 87
94 93 95 91 92
99 98 100 96 97
104 103 105 101 102
109 108 110 106 107
114 113 115 111 112
119 118 120 116 117
124 123 125 121 122
129 128 130 126 127
134 133 135 131 132
139 138 140 136 137
144 143 145 141 142
149 148 150 146 147
154 153 155 151 152
159 158 160 156 157
164 163 165 161 162
169 168 170 166 167
174 173 175 171 172
179 178 180 176 177
184 183 185 181 182
189 188 190 186 187
194 193 195 191 192
199 198 200 196 197
