# Supplementary material for: A High-Speed Visual BCI Based on Hybrid Frequency–Phase–Space Encoding and High-Density EEG Decoding
Source: Cyborg Bionic Syst. 2026 Mar 26;7:0555. doi: 10.34133/cbsystems.0555 (PMC13018654; doi:10.34133/cbsystems.0555)
Supplement: Supplementary 1 — Figs. S1 to S11 Tables S1 and S2 Movie S1 [file cbsystems.0555.f1.zip › SUPPLEMENTARY MATERIALS.docx]

SUPPLEMENTARY MATERIALS

***Fig. S1****. User interface of the 200-target BCI system and corresponding numeric labels for each target. (A) The user interface consisting of a text input box and 200 fixation points. (B) The numeric label corresponding to each fixation point. In the online experiment, this label is provided as feedback and displayed in the text input box at the top of the interface.*

***Fig. S2****. User interfaces of the 40, 80, 120, and 160 target BCI systems with optimal fixation combinations. (A) 40-target BCI system with fixation point of up. (B) 80-target BCI system with the fixation combination of down and up. (C) 120-target BCI system with the fixation combination of right, left, and up. (D) 160-target BCI system with the fixation combination of right, down, left, and up.*

***Fig. S3****. Paired t-test with Bonferroni correction showing significant performance differences between different electrode densities in the BCI systems. (A) Significance levels between different fixation combinations in the BCI systems with 40 (number of comparisons=10), 80 (number of comparisons=45), 120 (number of comparisons=45), and 160 (number of comparisons=10) targets. (B) Significance levels of classification accuracy for 4 electrode configurations (number of comparisons=6) with data lengths ranging from 0.1 to 0.5 seconds. (C) Significance levels of actual ITR for 4 electrode configurations (number of comparisons=6) with data lengths ranging from 0.1 to 0.5 seconds. (D) Significance levels of theoretical ITR for 4 electrode configurations (number of comparisons=6) with data lengths ranging from 0.02 to 0.1 seconds. A single asterisk, double asterisks, and triple asterisks indicate p<0.05, p<0.01, and p<0.001, respectively.*

***Fig. S4****. Effects of electrode density and decoding algorithm on classification performance. (A) Classification accuracy and (B) ITR of TDCA, TRCA, and EEGNet for classification tasks involving 40 to 200 targets (data length: 0.2 seconds) under 4 electrode configurations (9/64, 21/64, 32/128, and 66/256). Error bars indicate the standard errors (number of participants=15). Paired t-tests with Bonferroni correction were performed between decoding algorithms for each electrode configuration (number of comparisons=3). A single asterisk, double asterisks, and triple asterisks indicate p<0.05, p<0.01, and p<0.001, respectively.*

**

***Fig. S5****. Significance levels of actual ITR for 4 electrode configurations (number of comparisons=6) after personalizing the system parameters. A paired t-test with Bonferroni correction was performed. A single asterisk, double asterisks, and triple asterisks indicate p<0.05, p<0.01, and p<0.001, respectively.*

***Fig. S6****. Average SNR topographies at the fundamental and harmonic frequencies across 15 subjects for flicker stimuli with integer frequencies from 8 to 15 Hz. Each panel shows the SNR distribution for a single fixation position: (A) right fixation point, (B) left fixation point, (C) down fixation point, (D) up fixation point, and (E) center fixation point.*

***Fig. S7****. The phase topographies at the fundamental and harmonic frequencies for each subject in response to the 14 Hz flicker stimulus. Each panel shows the phase distribution for one fixation position: (A) right, (B) left, (C) down, (D) up, and (E) center fixation point.*

***Fig. S9****. Significance levels of accuracy for the electrode configurations under different number of electrodes. A paired t-test with Bonferroni correction (number of comparisons=3) was performed. A single asterisk, double asterisks, and triple asterisks indicate p<0.05, p<0.01, and p<0.001, respectively.*

***Fig. S8****. Significance levels of accuracy for 4 electrode configurations (number of comparisons=6) under the spatial information decoding and the frequency information decoding tasks. (A) Significance levels of accuracy for 4 electrode configurations under the 5-fixation-point classification tasks. (B) Significance levels of accuracy for 4 electrode configurations under the 40-flicker classification tasks. A paired t-test with Bonferroni correction was performed. A single asterisk, double asterisks, and triple asterisks indicate p<0.05, p<0.01, and p<0.001, respectively.*

***Fig. S10****. Comparison of decoding performance between the optimized 52/256 and full 66/256 electrode configurations and signal properties of discarded channels. (A) Classification accuracy for 40, 80, 120, 160, and 200 targets (data length: 0.2 seconds) using the optimized 52/256 and full 66/256 configurations. Paired t-tests were performed between different configurations. (B) Averaged SNR for each of the 66 channels, computed across 8 integer stimulation frequencies and 5 fixation points (corresponding to 40 targets in total). (C) Mean inter-channel correlation for each electrode, computed as the average correlation coefficient with all other channels. Electrodes discarded by the greedy search are highlighted in gray in panels B and C. Error bars indicate the standard errors (number of participants=15).*

***Fig. S11****. The optimal electrode layout for the optimal electrode number of 60 under the 200-target condition. Purple represents the selected electrodes, while white represents the unselected electrodes.*

***Table S1****. Target number and fixation combinations selected for each subject based on the principle of maximizing the actual ITR.*

| Subject | Target number | Fixation combination | Stimulation time (second) | Accuracy (%) | Theoretical ITR  (bps) | Actual ITR  (bpm) |
| --- | --- | --- | --- | --- | --- | --- |
| S1 | 160 | right down left up | 0.2 | 96.42 | 34.19 | 586.10 |
| S2 | 120 | right left up | 0.2 | 90.19 | 28.84 | 494.32 |
| S3 | 80 | right left | 0.2 | 92.57 | 27.36 | 469.01 |
| S4 | 80 | right left | 0.2 | 89.65 | 25.95 | 444.84 |
| S5 | 120 | right left up | 0.2 | 88.10 | 27.80 | 476.58 |
| S6 | 80 | down up | 0.2 | 94.10 | 28.13 | 482.25 |
| S7 | 80 | down up | 0.2 | 95.49 | 28.86 | 494.74 |
| S8 | 80 | down up | 0.2 | 84.10 | 23.44 | 401.78 |
| S9 | 160 | right down left up | 0.2 | 92.26 | 31.81 | 545.37 |
| S10 | 120 | right left up | 0.2 | 88.47 | 27.98 | 479.69 |
| S11 | 80 | down up | 0.2 | 85.56 | 24.08 | 412.77 |
| S12 | 80 | right left | 0.2 | 93.19 | 27.67 | 474.37 |
| S13 | 120 | right left up | 0.2 | 92.50 | 30.03 | 514.75 |
| S14 | 80 | down up | 0.2 | 90.42 | 26.31 | 451.04 |
| S15 | 160 | right down left up | 0.2 | 92.08 | 31.72 | 543.75 |
| Mean | — | — | — | — | 28.28 | 484.75 |
| STE | — | — | — | — | 0.74 | 12.68 |

***Table S2****. Encoding method, target number, accuracy, and actual ITR in different studies, as well as the data length, inter-trial interval, and whether a fixed or dynamic window was used in the classification strategy to compute the actual ITR.* *The table is arranged in chronological order from top to bottom, covering the years 2009 to 2024.*

| Study | Encoding method | Target number | Data length (second) | Trial interval (second) | Accuracy (%) | Actual ITR  (bpm) | Fixed or dynamic window |
| --- | --- | --- | --- | --- | --- | --- | --- |
| [22] | Frequency | 6 | 2 | 0.3 | 95.3 | 58 | Fixed |
| [21] | Time | 6 | 2.5 | 0.5 | 83 | 42.1 | Fixed |
| [20] | Code | 32 | 1.05 | 1.05 | 85 | 108 | Fixed |
| [23] | Phase | 4 | 1.5 | ― | 95.5 | 34.15 | Fixed |
| [19] | Code | 32 | 1.05 | 0.85 | 96.2 | 144.0 | Fixed |
| [18] | Frequency-time | 36 | 2.88 | 2 | 93.85 | 56.44 | Fixed |
| [17] | Frequency | 45 | 2 | 0.3 | 84.1 | 105 | Fixed |
| [3] | Frequency | 40 | 1.25 | 0.55 | 91.95 | 151.18 | Fixed |
| [16] | Frequency-phase | 40 | 0.5 | 0.5 | 91.04 | 264.79 | Fixed |
| [15] | Space | 9 | 3 | 1 | 95 | 40.8 | Fixed |
| [2] | Frequency-phase | 40 | 0.3 | 0.5 | 89.83 | 325.33 | Fixed |
| [14] | Frequency-phase | 40 | 0.22 | 0.5 | 88.2 | 353.3 | Dynamic |
| [13] | Space | 16 | 4 | 2~2.5 | 66.8 | 26.8 | Fixed |
| [12] | Frequency-phase-time | 108 | 1 | 0.7 | 81.67 | 172.46 | Fixed |
| [8] | Frequency-phase | 40 | 0.4 | 0.5 | 82 | 251.8 | Fixed |
| [10] | Frequency-space | 13 | 4 | 1~1.5 | 92.3 | 45.6 | Fixed |
| [11] | Frequency-time | 160 | 4 | 0.5 | 87.16 | 78.84 | Fixed |
| [9] | Frequency-phase | 40 | 1.27 | 0.5 | 92.7 | 164.72 | Dynamic |
| [6] | Frequency-phase | 120 | 0.7 | 1 | 92.47 | 213.23 | Fixed |
| [7] | Code | 120 | 0.52 | 0.52 | 76.58 | 265.74 | Fixed |
| [5] | Frequency-phase-time | 216 | 0.7 | 0.5 | 85.37 | 302.83 | Fixed |
| [4] | Space | 4 | 3 | 0.5 | 87.50 | 22.48 | Fixed |
| [1] | Frequency-phase-time | 40 | 0.2~ 0.3 | 0.5 | 95 | 366.05 | Fixed |

***Video S1****.* *Demo of a 200-target online experiment. Each trial consists of a 0.5-second cue period, during which one of the 200 fixation points turns red and the corresponding flicker is highlighted with a 15-pixel-wide red frame, followed by a 0.5-second stimulation period in which 40 flickers flash periodically and simultaneously. When the online analysis program returns a classification result, visual feedback—color changes of the selected fixation point and display of the corresponding numeric label in the text input box—is presented during the cue period of the next trial. In the actual online experiment, the number of targets and stimulation duration is individualized for each participant.*
