# Supplementary figures and images for: A High-Speed Visual BCI Based on Hybrid Frequency–Phase–Space Encoding and High-Density EEG Decoding
Source: Cyborg Bionic Syst. 2026 Mar 26;7:0555. doi: 10.34133/cbsystems.0555 (PMC13018654; doi:10.34133/cbsystems.0555)

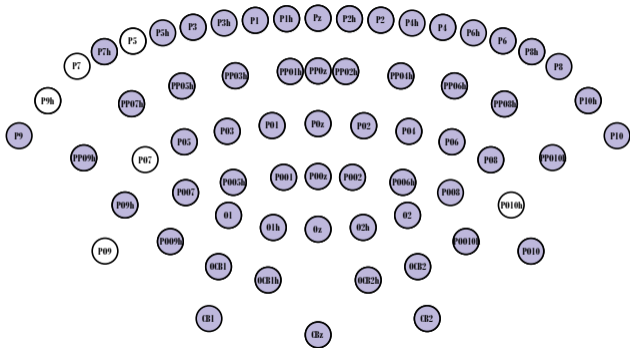

Supplement: Supplementary 1 — Figs. S1 to S11 Tables S1 and S2 Movie S1 [file cbsystems.0555.f1.zip › Fig.S11.pdf]

A

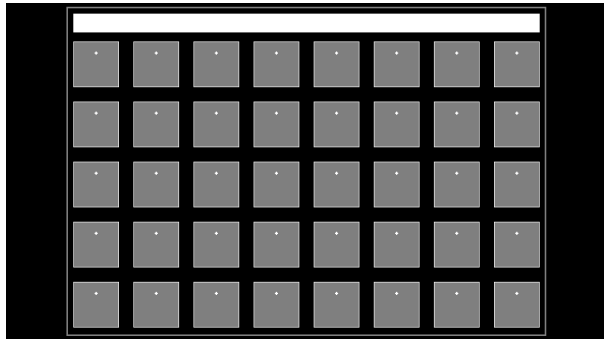

B

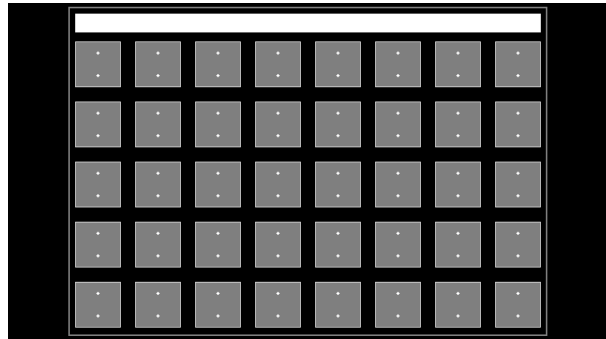

C

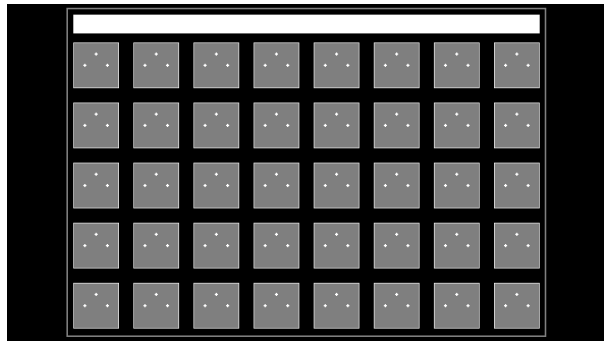

D

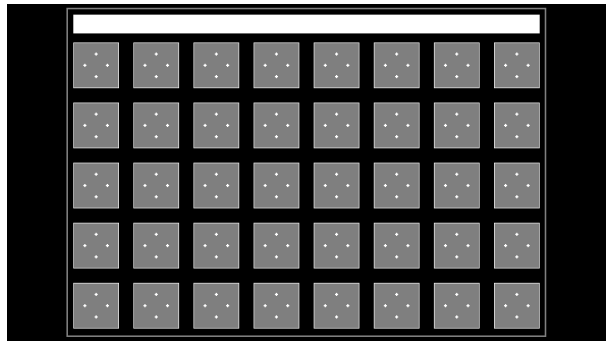

Supplement: Supplementary 1 — Figs. S1 to S11 Tables S1 and S2 Movie S1 [file cbsystems.0555.f1.zip › Fig.S2.pdf]

A

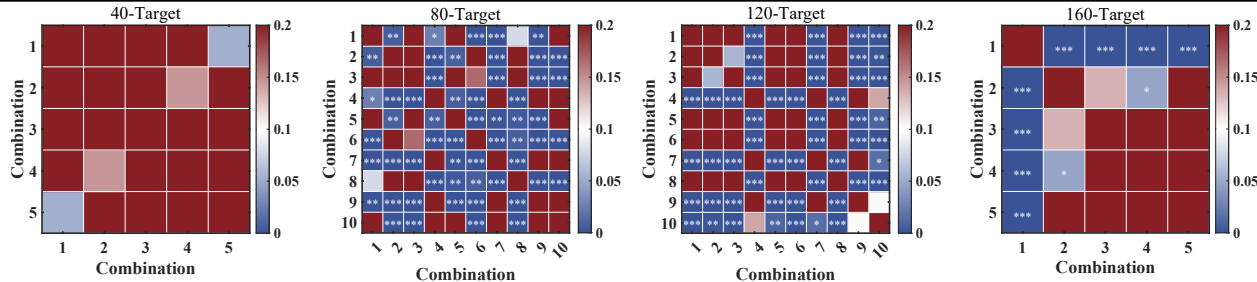

B

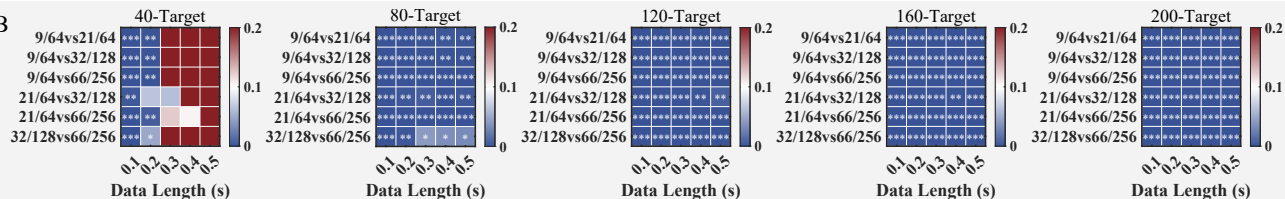

C

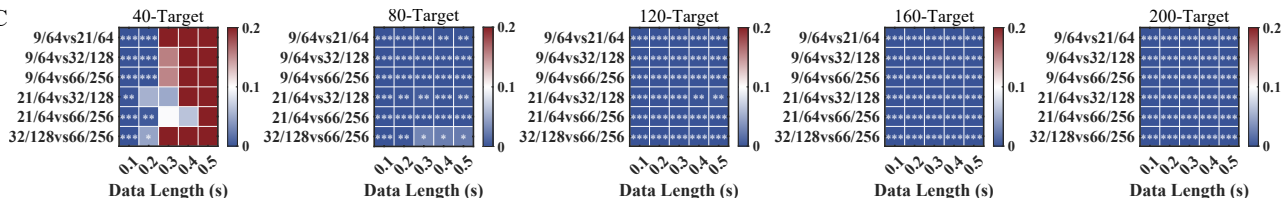

D

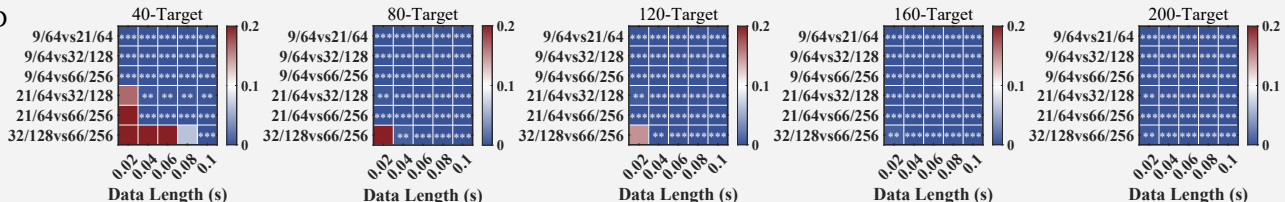

Supplement: Supplementary 1 — Figs. S1 to S11 Tables S1 and S2 Movie S1 [file cbsystems.0555.f1.zip › Fig.S3.pdf]

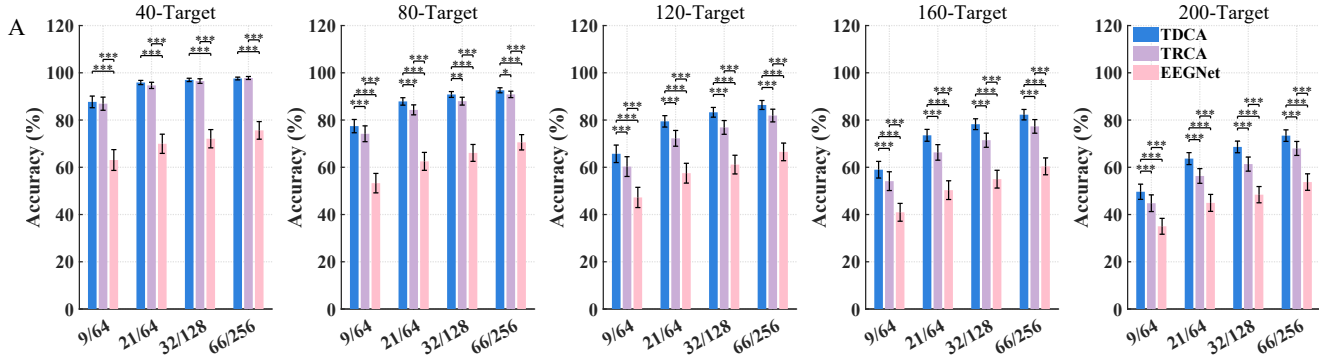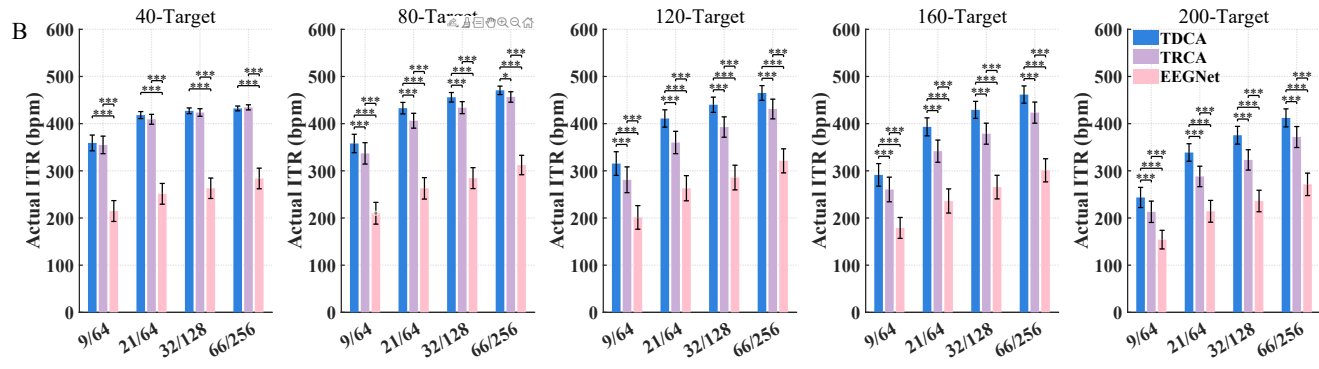

Supplement: Supplementary 1 — Figs. S1 to S11 Tables S1 and S2 Movie S1 [file cbsystems.0555.f1.zip › Fig.S4.pdf]

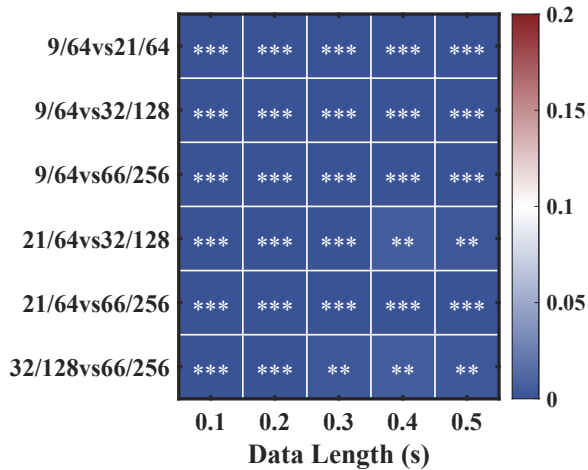

Supplement: Supplementary 1 — Figs. S1 to S11 Tables S1 and S2 Movie S1 [file cbsystems.0555.f1.zip › Fig.S5.pdf]

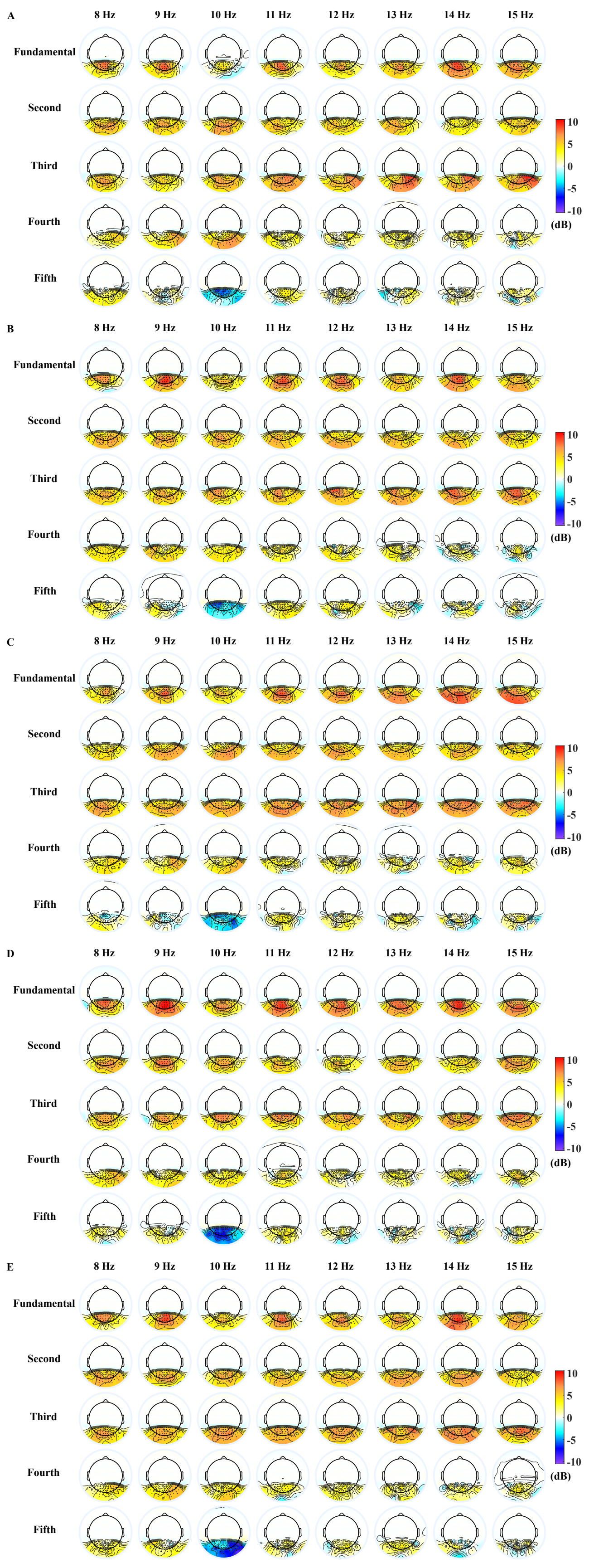

Supplement: Supplementary 1 — Figs. S1 to S11 Tables S1 and S2 Movie S1 [file cbsystems.0555.f1.zip › Fig.S6.pdf]

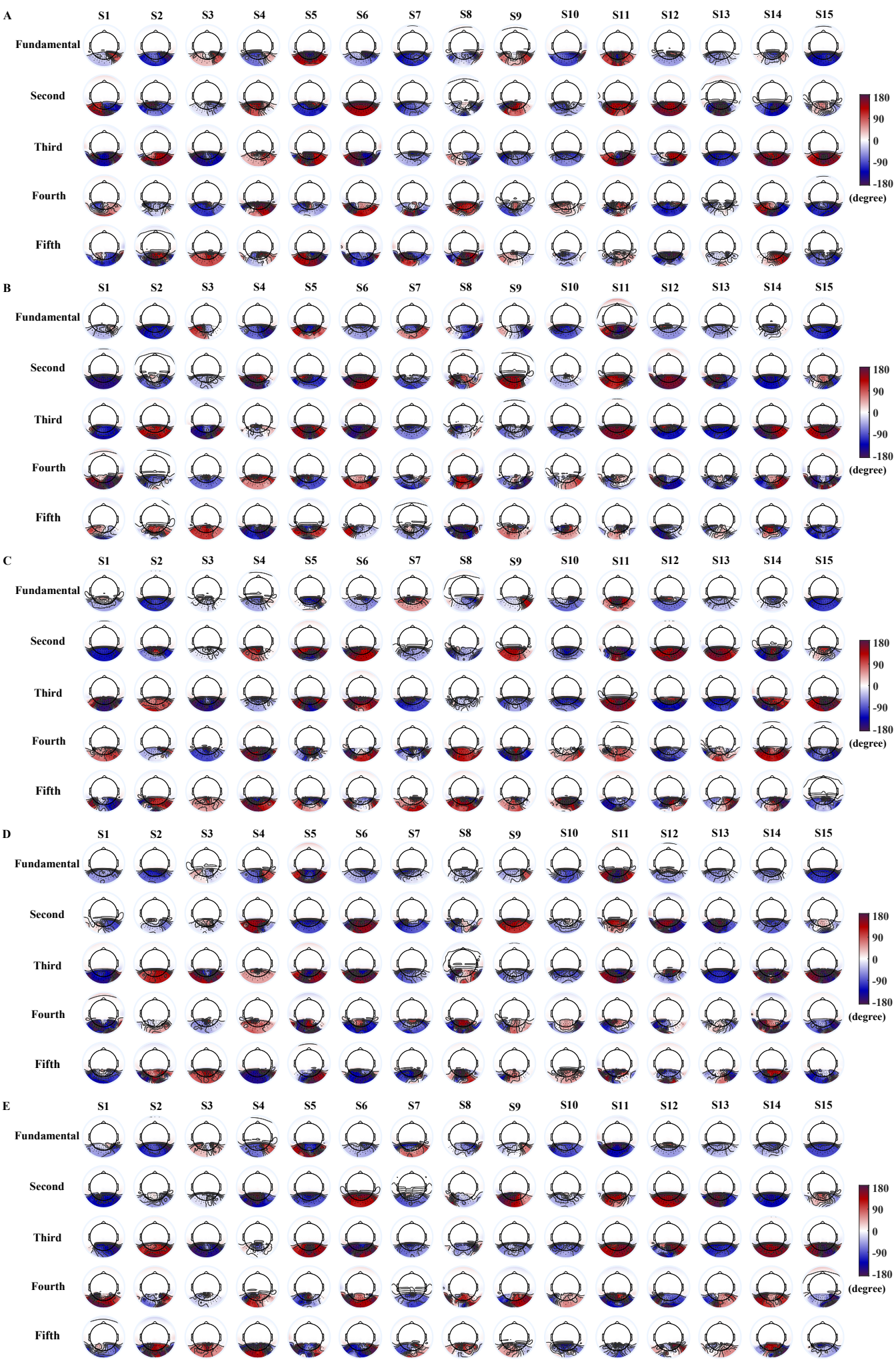

Supplement: Supplementary 1 — Figs. S1 to S11 Tables S1 and S2 Movie S1 [file cbsystems.0555.f1.zip › Fig.S7.pdf]

A

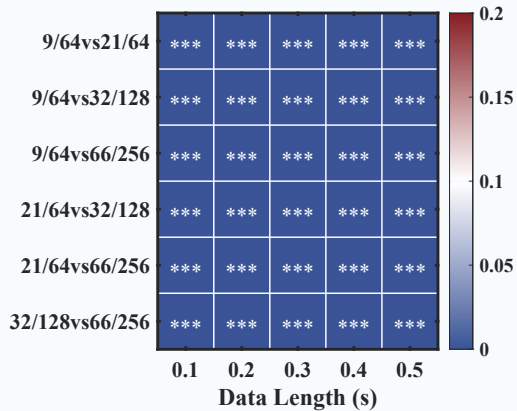

B

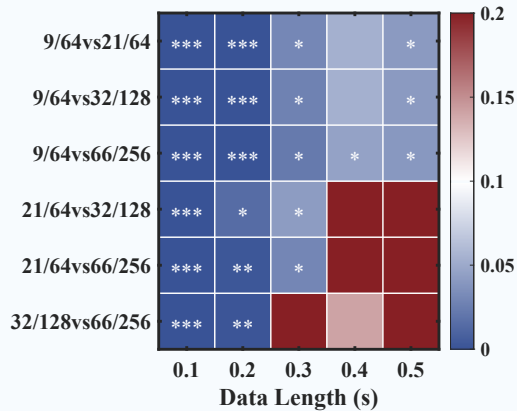

Supplement: Supplementary 1 — Figs. S1 to S11 Tables S1 and S2 Movie S1 [file cbsystems.0555.f1.zip › Fig.S8.pdf]

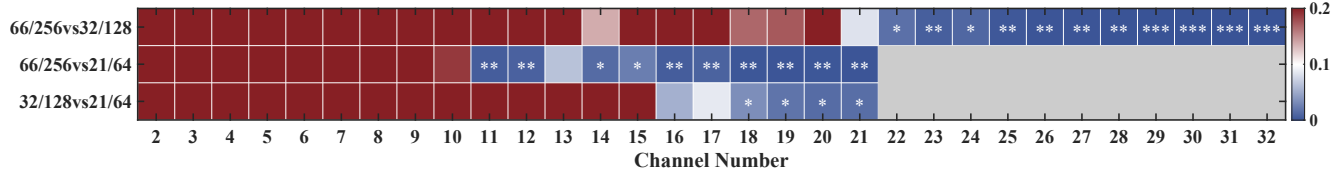

Supplement: Supplementary 1 — Figs. S1 to S11 Tables S1 and S2 Movie S1 [file cbsystems.0555.f1.zip › Fig.S9.pdf]
