# Supplementary material for: A High-Speed Visual BCI Based on Hybrid Frequency–Phase–Space Encoding and High-Density EEG Decoding
Source: Cyborg Bionic Syst. 2026 Mar 26;7:0555. doi: 10.34133/cbsystems.0555 (PMC13018654; doi:10.34133/cbsystems.0555)
Supplement: Supplementary 1 — Figs. S1 to S11 Tables S1 and S2 Movie S1 [file Fig.S10.pdf › Fig.S10.pdf]

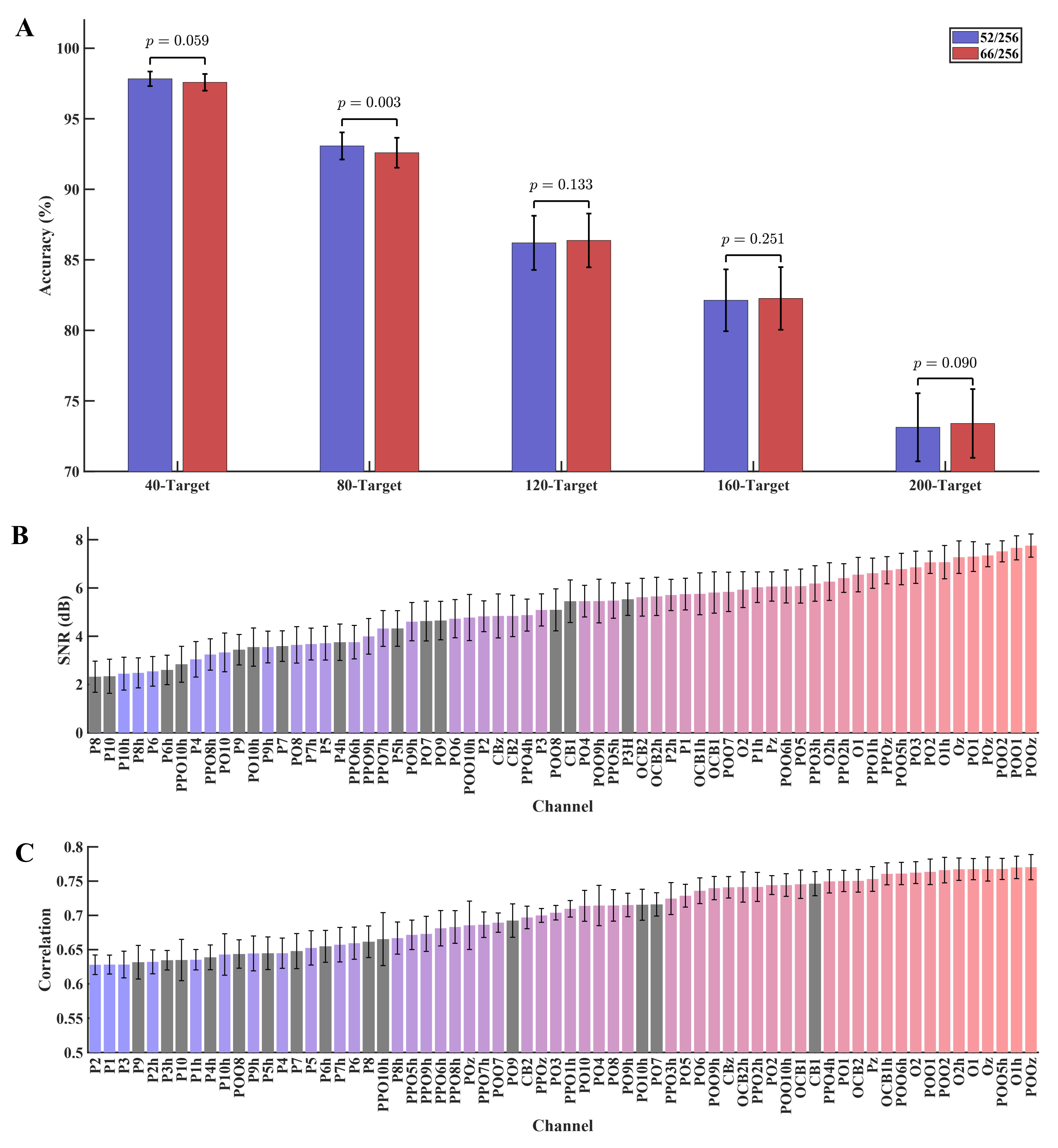

A
52/256
66/256
Accuracy (%)
$p = 0.059$
$p = 0.003$
$p = 0.133$
$p = 0.251$
$p = 0.090$
100
95
90
85
80
75
70
40-Target
80-Target
120-Target
160-Target
200-Target
B
SNR (dB)
8
6
4
2
0
P8 P10 P10h P8h P6 P6h PPO10h P4 PPO8h PO10 P9 PO10h P9h P7 PO8 P7h P5 P4h PPO6h PPO9h PPO7h P5h PO9h PO7 PO9 PO6 POO10h P2 CBz CB2 PPO4h P3 POO8 CB1 PO4 POO9h PPO5h P3H OCB2 OCB2h P2h P1 OCB1h OCB1 POO7 O2 P1h Pz POO6h PO5 PPO3h O2h PPO2h O1 PPO1h PPOz POO5h PO3 PO2 O1h Oz PO1 POz POO2 POO1 POOz
Channel
C
Correlation
0.8
0.75
0.7
0.65
0.6
0.55
0.5
P2 P1 P3 P9 P2h P3h P10 P1h P4h P10h POO8 P9h P5h P4 P7 P5 P6h P7h P6 P8 PPO10h P8h PPO5h PPO9h PPO6h PPO8h POz PPO7h POO7 PO9 CB2 PPOz PO3 PPO1h PO10 PO4 PO8 PO9h PO10h PO7 PPO3h PO5 PO6 POO9h CBz OCB2h PPO2h PO2 POO10h OCB1 CB1 PPO4h PO1 OCB2 Pz OCB1h POO6h O2 POO1 POO2 O2h O1 Oz POO5h O1h POOz
Channel
